# Immunomodulatory Molecular Mechanisms of *Luffa cylindrica* for Downy Mildews Resistance Induced by Growth-Promoting Endophytic Fungi

**DOI:** 10.3390/jof8070689

**Published:** 2022-06-29

**Authors:** Mamoona Rauf, Asim Ur-Rahman, Muhammad Arif, Humaira Gul, Aziz Ud-Din, Muhammad Hamayun, In-Jung Lee

**Affiliations:** 1 Department of Botany, Garden Campus, Abdul Wali Khan University Mardan, Khyber Pakhtunkhwa, Mardan 23200, Pakistan; mamoona@awkum.edu.pk (M.R.); asimurrahman1@gmail.com (A.U.-R.); gulhumaira@awkum.edu.pk (H.G.); 2 Department of Biotechnology, Garden Campus, Abdul Wali Khan University Mardan, Khyber Pakhtunkhwa, Mardan 23200, Pakistan; 3 Department of Biotechnology and Genetic Engineering, Hazara University, Mansehra 21120, Pakistan; geneticsaz@gmail.com; 4 Department of Applied Biosciences, Kyungpook National University, Daegu 41566, Korea

**Keywords:** *Luffa cylindrica*, *Pseudoperonospora cubensis*, downy mildew, *Trichoderma virens*, *Trichoderma harzianu*, endophytic fungi, biocontrol

## Abstract

Downy mildew (DM), caused by *P. cubensis*, is harmful to cucurbits including luffa, with increased shortcomings associated with its control through cultural practices, chemical fungicides, and resistant cultivars; there is a prompt need for an effective, eco-friendly, economical, and safe biocontrol approach. Current research is therefore dealt with the biocontrol of luffa DM1 through the endophytic fungi (EF) consortium. Results revealed that *T. harzianum* (ThM9) and *T. virens* (TvA1) showed pathogen-dependent inducible metabolic production of squalene and gliotoxins by higher gene expression induction of *SQS1/ERG9* (*squalene synthase*) and *GliP* (*non-ribosomal peptide synthetase*). Gene expression of lytic enzymes of EF was also induced with subsequently higher enzyme activities upon confrontation with *P. cubensis*. EF-inoculated luffa seeds showed efficient germination with enhanced growth potential and vigor of seedlings. EF-inoculated plants showed an increased level of growth-promoting hormone GA with higher gene expression of *GA2OX8*. EF-pre-inoculated seedlings were resistant to DM and showed an increased GSH content and antioxidant enzyme activities (SOD, CAT, POD). The level of MDA, H_2_O_2,_ REL, and disease severity was reduced by EF. ACC, JA, ABA, and SA were overproduced along with higher gene expression of *LOX*, *ERF*, *NCED2*, and *PAL*. Expression of defense-marker genes (*PPO*, *CAT2*, *SOD*, *APX*, *PER5*, *LOX*, *NBS-LRR*, *PSY*, *CAS*, *Ubi*, *MLP43*) was also modulated in EF-inoculated infected plants. Current research supported the use of EF inoculation to effectively escalate the systemic immunity against DM corresponding to the significant promotion of induced systemic resistance (ISR) and systemic acquired resistance (SAR) responses through initiating the defense mechanism by SA, ABA, ET, and JA biosynthesis and signaling pathways in luffa.

## 1. Introduction

Phytopathogenic fungi have been imposing severe losses to global food security. The DM pathogen, *Pseudoperonospora cubensis* (Berk. & Curt.) Rost. (*Oomycota, Peronosporaceae*), is an obligate biotroph that attacks over 40 cucurbitaceous host species (about 20 genera). Infection occurs on cotyledons and true leaves [1]. Luffa, the sponge gourd (*Luffa cylindrica* (L.) M. Roem.; syn. *Luffa aegyptiaca*), is grown mainly in Asia by smallholder farmers due to its nutritional importance. Luffa fruits contribute potassium, calcium, magnesium, and vitamin A (139, 20, 14 mg/100 g fresh weight, and 410 IU, respectively) to the human diet [2]. DM is a crucial biotic constraint to luffa cultivation throughout Asia, and controlling this pathogen is a major challenge for luffa growers. Due to the time lag taken to breed hybrids and the capacity of the pathogen for controlling the resistance machinery of the plant, an alternative approach should be adapted to modulate the available cultivars, which can promote the growth of the plant and improve its defense mechanism against pathogens. Moreover, because of the hazardous impact of fungicide on the human health and the environment, researchers are exploring non-chemical and eco-friendly alternative approaches.

In the natural environment, plants develop responses provoked by biocontrol agents (BCAs) to cope with biotic stresses. Thus, the use of BCAs for exploiting their secondary metabolites (SMs) is potentially a useful strategy. These responses have two major phyto-immune systems that include the SAR and the ISR. Some BCAs control phytopathogens by indirect interactions through activating the ISR, which is a contrasting mechanism to the well-studied mechanism of SAR induced by a pathogen’s attack [3]. *Trichoderma* are reported to (i) be effective biocontrol agents against fungal phytopathogens; (ii) be effective biofertilizer and growth stimulators; (iii) induce abiotic stress and biotic resistance; and (iv) secrete chemical elicitors for stress response induction in plants. *Trichoderma* (*T. virens, T. harzianum*, and *T. asperellum*) are well-known BCAs used globally because they produce a plethora of antimicrobial SMs [4].

The previous report showed that host genotype and genetic variability between different lines also contribute to determining the outcome of interactions with plant growth-promoting microbes, BCAs, and pathogens [5]. Several *Trichoderma* strains are known as BCAs against phytopathogens, such as *P. cubensis, B. cinerea, S. fusca, S. sclerotium, P. capsici, P. citrophthora, P. megakarya, P. palmivora, P. ultimum*, and *P. erythroseptica*. *T. hamatum* (T382) efficiently enhanced the ISR mechanism against the pathogen (*B. cinerea*) of *Arabidopsis* [6] and *X. vesicatoria* in tomato [7]. *T. hamatum* (GD12) induced resistance in rice against *M. oryzae*, *S. lettuce* against *sclerotiorum* and *R. solani*. *T. harzianum* (Th10) triggered resistance in pepper against *P. capsica. T. roseum* triggered resistance in chickpea against *M. phaseolina*. *T. harzianum* strain T-22 induced tolerance in tomatoes against cucumber mosaic virus [8,9].

Particularly for DM, the *T. harzianum* (T39) acted by eliciting ISR against DM (*P. viticola*) of grapevines, as was also observed in cucumber against powdery mildew disease. It also protected grapevines against DM infection in a greenhouse [10]. *T. harzianum* (PGPFYCM-8, PGPFYCM-2, PGPFYCM-14) enhanced the growth of the plant and triggered tolerance against *P. halstedii*, the causative agent of sunflower DM [11]. *T. atroviride* (TRS25) has triggered DM resistance by systemic defense responses in cucumbers. DM resistance in pearl millet was found by systemic immunity upon inoculation of *T. hamatum* strain UoM13 isolated from pearl millet host plants [12]. *T. brevicompactum* (UP-91) elicited systemic resistance in pearl millet DM disease [13]. *T*. *asperellum* (T34), *T. harzianum* (T39), and *T. atroviride* (SC1) induced grapevine resistance against DM by producing volatile organic compounds (VOCs) [14]. Current knowledge suggests the initiation of differential induction of ISR depends on a specific strain producing a specific variety of metabolites.

*Trichoderma* secretes metabolites that play a role in the biological control of diseases. Several *Trichoderma* have shown anti-phytopathogenic activity by producing diverse antifungal SMs and several proteinaceous elicitors. *Trichoderma* has also been reported to induce the ISR by regulating defense-related gene expression, such as Sm1 (*T. virens*) activated the ISR in maize [15]. The effect of *T. harzianum* (Epl-1) protein has also been found in regulating the *Botrytis* infection in tomatoes by inducing defense-related genes [16].

Many growth-promoting *Trichoderma* produce SMs with strong antimicrobial activity against phytopathogenic fungi. For example, harzianolide and harzianic acid (a tetrameric acid with siderophore-like activity) promoted the growth as well as inhibited diverse phytopathogens, including *P. irregulare, R. solani*, and *S. sclerotiorum* [17]. *Trichoderma*-mediated ISR stimulation in hosts is known to be as efficient as that found by chemicals or fungicides. Cell-free culture filtrates (CF) of *Trichoderma* have enhanced SAR in oilseed rape against pathogens by stimulating signaling molecules. *T. harzianum* TH12 and its CF also proved to be effective growth inhibitors against the phytopathogen *S*. *sclerotiorum* [18]. *T. harzianum* Epl-1 protein content was also effective against *Botrytis* attack on tomato and initiated the expression of the defense-related genes through elicitation of the salicylic acid pathway [16].

*Trichoderma* is also known to efficiently show antifungal potential against phytopathogenic fungi by producing different groups of SMs, namely Epipolythiodioxopiperazines (ETPs) such as gliotoxin, ergosterol derivatives, and precursors (squalene), butenolides, pyridones, peptaibols, azaphilones, pyrones, steroids, koninginins, lactones, trichothecenes, anthraquinones, viridins, heptelidic acid, harzialactones and derivatives, azaphilones, trichodermamides, nthraquinones, butenolides, isocyano metabolites, daucanes, acoranes, diketopiperazines, viridiofungins, cyclopentenone derivatives, cyclonerodiol derivatives, statins, bisorbicillinoids, setin-like metabolites, and nitrogen heterocyclic [15].

The role of ergosterol in eliciting plant defense response along with the involvement of squalene (ergosterol precursor) indicates a balance between squalene conversion to ergosterol. This metabolite is essential for not only maintaining cellular membrane stability but also helps in establishing interactions with plants [19,20] to modulate the defense-related gene expression upon fungal pathogen attack [21]. In addition, ergosterol-elicitation responses have also been shown to induce JA signaling [22]. Moreover, the sulfur-containing mycotoxins (gliotoxins) are produced by several fungal species including *T. virens* (*G. virens*) and are well-known for their antiviral, antifungal, and immunomodulating properties, particularly in *T. virens* [23]. To date, the exact modes of action of gliotoxins for triggering antagonism of fungal cells are not well-explored. Chemical communication through secreted SMs of *Trichoderma* for root colonization induces signals transmission by modulating the production of SA, JA, ET, ABA, or reactive oxygen species (ROS) that influence gene expression of defense-related proteins such as lytic enzymes. Generally, antibiotic activity in cooperation with lytic enzymes is a double-action that proposes a complex level of antagonistic activity.

*Trichoderma* interaction in luffa and its role in DM suppression has not yet been studied so far. Further studies are required to identify growth-promoting *Trichoderma* isolates that will be capable of eliciting disease tolerance against DM of luffa by inhibiting the pathogenic fungi. Such antagonistic *Trichoderma* isolates may also be integrated with safer fungicides to enhance disease control in farmland. To this end, the present study is aimed to induce resistance against DM by exploitation of the endophytic *Trichoderma* consortium and to explore its ability to drive the molecular triggers for suppressing the pathogenicity of *P. cubensis* in luffa.

## 2. Materials and Methods

### 2.1. Endophytic Isolation, Characterization, and Growth-Promotion Plant Bioassays

***Endophytic Fungal Isolation*.** For the isolation of fungal endophytes, roots of *Canna indica* L. were obtained from the stream banks Tarbela lake (Swabi) built on Indus River of Pakistan, with north latitudes (33°/55′ and 34°/23′) and east longitudes (72°/13′ and 72°/49′). The method was adapted from [24] for root segments sterilization, fungal endophytic isolation, and scaling up the endophytic fungal cultures that were used for further analysis to characterize the fungal isolates.

***Identification of M9 and A1 Isolates*.** DNA extraction was performed from the individual strains (M9 and A1) for which DNeasy plant mini kit (QIAGEN, Valencia, CA, USA) was used. Identification of fungal isolates was performed by amplifying internal transcribed regions (ITS) of 18S rDNA. The universal primers (*ITS1* and *ITS4*) amplified the *18S rDNA* (*ITS1-5.8S-ITS2*) [25] and sequenced for preliminary identification. Sequencing was performed by BGI Co., Ltd. (Shenzhen, China) using universal primers (*ITS1* and *ITS4*). The ITS sequence was classified to the species level after a homology search on GenBank that revealed the highest similarity of M9 with *T. harzianum* and A1 with *T. virens*. Hence, the strain M9 was identified as *T. harzianum* (ThM9) and A1 as *T. virens* (TvA1).

Specific and differential identification of fungal isolates was performed by using specific primer pairs *tef1-F* and *tef1-R* for TvA1 and RPB2-F and RPB2-R for ThM9, which were selected for specific amplification by PCR using 10–50 ng of gDNA from ThM9 and TvA1 PCR products were visualized, purified, and sequenced for phylogenetic analysis. To infer the phylogenetic status for fungal endophytes identification, the sequence comparison was performed by BLAST search analysis from NCBI database http://www.ncbi.nlm.nih.gov/, accessed on 22 April 2022). The phylogenetic tree construction was done by neighbor -joining (NJ) and maximum likelihood (ML) protocols from MEGA X software, and 1 K-bootstrap replication were used as statistical support. Sequences were submitted to NCBI GenBank under accession no. ON315869 for *T. harzianum* (ThM9) and ON315868 for *T. virens* (TvA1).

***Characterization of the ThM9 And TvA1 for Plant Growth-Promoting Traits*.** The GA concentration was determined using the method described by [26]. Quantification of protein concentration was performed by (Bradford, 1976). IAA estimation was done by Salkowski reagent according to the protocol of [27]. 2,2-Diphenyl-1-picrylhydrazyl (DPPH) free radical scavenging activity was quantified as mentioned by [28]. Proline content was determined using the procedure of (Bates, Waldren, and Teare, 1973). Total phenols and flavonoids were quantified as described by [29]. The endophytic fungal-root association was quantified by calculating root colonization frequency (% CF) as described by [30].

***Trichoderma Isolates Compatibility Bioassay*.** Both *Trichoderma* isolates did not over-grow and restricted the growth of mycelium and sporulation, indicating the compatibility to be used as a consortial combination for possible enhancement of growth in plants bioassays.

***Application of ThM9 and TvA1 for Plant-Germination and Growth Bioassay*.** Conidial suspensions of *ThM9* and *TvA1* strains were obtained by harvesting the biomass from growth media. Conidial density was determined using a hemocytometer. Fifty surface-sterilized luffa seeds were soaked for each treatment in 1 mL (10^8^ conidia/mL suspension) of each *ThM9* and *TvA1* strain individually as well as in a consortial combination. Inoculated seeds were air-dried in a laminar flow cabinet. Control seeds were soaked in sterile water (1 mL) under the same conditions. One plant/pot was placed in the controlled environment of a greenhouse (humidity 75%, photoperiod 16 h, temperature 18–28 °C).

The experiment was carried out under four different conditions:

(1) Control;
(2) ThM9;
(3) TvA1;
(4) TvA1 + ThM9.

### 2.2. Pathogen Isolation, Characterization, and Plant Pathogenicity Bioassay

***Source and Maintenance of the Pathogen*.** A single-lesion isolate of *P. cubensis* was obtained from luffa leaves from Mardan, Pakistan, from July–August 2019 to 2020 and maintained by propagating it on the leaves of healthy luffa plants in the laboratory. The leaves from 5-week-old plants were placed on moist paper towels in acrylic boxes. The abaxial side of the leaf was inoculated with conidial suspension (10^8^ conidia/mL). Inoculated leaves were then covered and incubated in controlled conditions at 24/18 °C for 16/8 h (day/night) with humidity of 60% during the day and 80% at night.

***Specie-Specific Identification of DM Causative Pathogen*.** The method of [31] was adapted for DNA extraction from DM-causative pathogen (DM1). Specie identification of pathogen isolate was done by using species-specific primer pairs (*cox2-F* and *cox2-R-clade1*) and (*cox2-F* and *cox2-R-clade1/2*), which is specific for mitochondrial marker (*cox2*) gene (Table 1) by using nested PCR method reported by [32]. Sequencing was done by BGI Co., Ltd. (Shenzhen, China), and phylogenetic analysis by maximum likelihood and neighbor-joining method from MEGA X software and 1 K-bootstrap replication were used as statistical support. DM1 was identified as *P. cubensis*, and the sequence was submitted to NCBI GenBank under accession no. ON243884.

***Leaf-Dip Confrontation Assay for Mycoparasitism*.** The potential of ThM9 and TvA1 isolates for the synthesis of plant-growth-promoting and antifungal-bioactive compounds was evaluated by establishing an endophyte–pathogen confrontation assay (infected leaf dip culture assay) in a time-course manner. There were two treatments for each in this experiment:

(1) ThM9 (control); (2) ThM9 × *P. cubensis*;

(1) TvA1 (control); (2) TvA1 × *P. cubensis*.

To this end, the detached whole luffa leaves heavily infected with the pathogen were dipped into the 100 mL of Czapek culture broth media pre-inoculated with 10 µL (1 × 10^8^ CFU mL^−1^) of the conidial suspension of endophytic fungi.

### 2.3. In Vitro Screening of Fungal Endophytes for Anti-Fungal Enzymes and Metabolites

***Inductionof Antifungal Enzymes*.** Time-course induction of defense-related enzyme activities in ThM9 and TvA1 endophytic fungi was evaluated by co-inoculating the 100 mL Czapek culture broth media with 10 µL conidial suspension (1 × 10^8^ CFU mL^−1^) from each of the ThM9 and TvA1 endophytic fungal culture and conidial suspension of *P. cubensis* (1 × 10^8^ conidial mL^−1^) according to the protocol of Zhang [33]. The freshly prepared spore suspension was used in all experiments.

***Extraction of Antifungal Enzymes*.** Fresh leaf tissue (1 g) from luffa plants was harvested and ground with extraction buffer (1 mL) and centrifuged (12,000× *g*/20 min/4 °C). The supernatant was used for the calculation of enzymatic activities by Lowry’s method using BSA (Sigma) as a standard [34].

***Quantification of Antifungal Enzymatic Activities*.** β-1,3-Glucanase enzyme extraction was performed by adding sodium acetate buffer (50 mM, pH 5.2). Specific enzyme activity was assessed by the protocol [35]. The Chitinase activity was assessed by the method of Rodriguez-Kabana [36]. The cellulase assay was carried out by adapting the procedure described by [37]. Protease activity was estimated by adding azocasein (A 2765 Sigma Co., St. Louis, MO, USA) as described earlier [38].

***Quantification of Ergosterol and Squalene*.** The metabolic extraction of intracellular and secreted ergosterol and squalene was performed by using fungal biomass and culture filtrate (CF) as reported by [39,40]. To this end, the pure fungal colony was cultured in 100 mL Czapek growth media and placed in a 120 rpm shaker incubator (30 °C temperature). After 24, 48, 72, and 96 h of growth, the pellet of biomass was harvested by centrifugation (4000× *g*/4 °C/15 min), lyophilized, and stored at −70 °C for quantification of intracellular ergosterol and squalene content, while CF was harvested for quantification of secreted ergosterol and squalene content. All measurements were made in triplicate.

### 2.4. In Planta Screening for Endophytic Fungal Antagonism against DM

***Plant Growth Environment*.** Seeds were sterilized and incubated to germinate for 6 days, then transplanted to the soil pots and subjected to growth under a controlled environment (16/8 h day/night, 25/18 °C, humidity 75%, white light intensity 150–220 μmol m^−2^ s^−1^). Thirty-five-day-old plants were used for the leaf biocontrol assays.

***Preparation of P. cubensis Spore Suspension*.** As *P. cubensis* is a non-culturable fungus and cannot be grown on an artificial nutrient medium. To collect the sporangia, the heavily infected leaves were obtained from luffa plants affected by the DM disease in a greenhouse. Spores from lesion areas of infected leaves were collected by spraying autoclaved ddH_2_O added with 0.01% Triton X-100. The spore suspension was spun (Beckman J2-HS) at 10,000× *g* for 15 min. Pellets were washed 3X with sterile dH_2_O, and vertexing was done for 30 s. Resuspension of the pellet was done in sterile dH_2_O at a final concentration of 1 × 10^8^ spores/mL. The spore suspension was monitored by a hemocytometer (Olympus BX53 Upright Microscope) was used for spore density check.

***Bioassay for Leaf Disk and Whole Detached Leaf*.** Healthy leaves were detached (3rd to 6th node of luffa plants) and washed, and leaf disks (diameter: 0.5 inches) were generated with aid of a puncher. Flame sterilized forceps were used to place the leaf discs into the sterilized Petri plates for soaking them in fungal spore suspension in Czapek media (1 × 10^8^ CFU/mL). Six discs for each treatment were placed on a damp filter paper (Whatman No. 1/5.5 cm) soaked with growth media (2 mL) of composition previously described before [41]. The leaf disc samples were kept at 30 °C for 24 h in dark followed by the application of freshly prepared conidial suspension (10 μL) on the center of leaf disk tissue as a pathogen inoculum. Sealed Petri plates were incubated in controlled conditions (21/18 °C under 12 h/12 h light/dark cycle). In the whole detached leaf bioassay, healthy leaves of luffa plants were detached, washed, and transferred onto a filter paper soaked with the nutrient media.

Leaf disk and whole detached leaf bioassay were done thrice, independently, as separate biological replicates with at least six technical replicates for each treatment. For control, the equivalent volume of nutrient solution and Czapek media was supplied.

There are eight treatments included in this experiment:

(1) Control = nutrient solution + Czapek media;
(2) Endophytic fungus 1 = ThM9;
(3) Endophytic fungus 2 = TvA1;
(4) Endophytic fungus 1 + 2 = TvA1+ThM9;
(5) Control = nutrient solution + *P. cubensis*;
(6) Endophytic fungus 1 + pathogen = ThM9 + *P. cubensis*;
(7) Endophytic fungus 2 + pathogen = TvA1 + *P. cubensis*;
(8) Endophytic fungus 1 + 2 + pathogen = TvA1 + ThM9 + *P. cubensis*.

***Disease severity Quantification***. Disease severity was analyzed by setting 0–7 grades that were assigned based on chlorosis and development of infected lesions. Disease severity was assayed by cross-examining infected leaf disc photos corresponding to differential levels of infection symptoms. The percentage for the disease index was quantified by the formula given:

Disease index (%) = ΣDisease Grade × The number of leaves in this grade/total leaves of the plant with the highest disease grade

The severity grading for DM infection on luffa leaf discs was followed as described previously [42].

Grade 0: leaves (disease spots absent);
Grade 1: Disease spots (<5%/total leaf area);
Grade 2: Disease spots (6–10%/total leaf area);
Grade 3: Disease spots (11–25%/total leaf area);
Grade 4: Disease spots (26–50%/total leaf area);
Grade 5: Disease spots (50–60%/total leaf area);
Grade 6: Disease spots (60–70%/total leaf area);
Grade 7: Disease spots (>70%/total leaf area).

### 2.5. Characterization of Plants for Growth Promotion and Pathogen Resistance

***DAB and trypan blue histochemical analysis*.** For monitoring the H_2_O_2_ production and accumulation with DAB (3,3′-diaminobenzidine; Sigma, St. Louis, MO, USA), luffa leaf tissues were exposed to DAB staining solution by soaking the luffa leaf tissues in DAB solution (1 mg·mL^−1^) and kept for 8 h. Tissues then were boiled for 20 min in ethanol/lactic acid/glycerol (3:1:1 ratio) and transferred to ethanol (95%) at 4 °C for the store. H_2_O_2_ was visualized and assayed by a brown color appearance due to polymerization of DAB. Images from leaf segments were taken with the aid of a digital camera (Canon). Histochemical bioassay was performed in triplicates for each condition mentioned in the leaf disc bioassay. Endogenous H_2_O_2_ content was quantified by grinding the leaf tissues in liquid nitrogen, and sodium phosphate buffer (pH 6.5/20 mM/1 mL) was quickly added to the ground sample (30 mg) and mixed well. The extraction was centrifuged (10,000× *g*/10 min/4 °C), and the supernatant was used for measuring absorbance (410 nm) as mentioned earlier [43]. The cell death of infected luffa leaf tissues was detected by trypan blue staining by immersing the treated leaf tissues in trypan blue solution. Samples were boiled for 10 min and placed at 37 °C overnight. Stained leaf tissues were decolorized and photographed by microscopy.

***Determination of electrolyte Leakage and malondialdehyde*** (***MDA***) ***content*.** Content for MDA in luffa leaves was quantified as described by (Heath and Packer, 1968), and electrolyte leakage was quantified as (C_i_/C_max_) × 100.

***Physiological Analysis*.**

Leaf chlorophyll content was monitored through a chlorophyll meter (SPAD-502, Minolta, Corp., Spectrum Technologies, Aurora, IL, USA). The stomatal conductance (*g_s_*) was assessed by exposing the abaxial surface of the leaf to the leaf porometer (Decagon Devices, Inc., Pullman, WA, USA).

***Extraction of antioxidant enzymes*.** The spectrophotometric method was used to quantify the specific enzymatic activities. The reduced glutathione (GSH) content was measured as described previously [44]. Superoxide dismutase (SOD) enzyme activity was quantified by measuring its inhibition potential against the photochemical reduction of nitroblue tetrazolium (NBT) [45]. Peroxidase (POD) activity was determined according to the protocol of Gorin and Heidema [46]. Ascorbate peroxidase (APX) enzyme activity was measured through the oxidation of ascorbic acid by the protocol of Asada [47]. Catalase (CAT) enzyme activity was quantified as mentioned by Chandlee and Scandalios [48]. 

***Phytohormonal analysis*.** Leaves from the 3rd to 6th node of luffa plants were detached for measuring the endogenous level of ethylene (ET) precursor; 1-Aminocyclopropane 1-Carboxylic Acid (ACC), abscisic acid (ABA), salicylic acid (SA), and jasmonic acid (JA) contents from each condition.

The ET production was monitored by quantifying the endogenous ACC content as reported by Yu [49].

ABA was quantified as mentioned by Perata [50]. First, 150 mg of frozen leaf tissue was powdered with HPLC-grade H_2_O (0.8 mL), followed by an overnight incubation (4 °C). Sonication, centrifugation (16,000× *g*/10 min/4 °C), and filtration (0.2 μm Minisart SRT 15 filters) were performed to harvest the supernatant for quantification. HPLC separation was done by using a reverse-phase Dionex column (Acclaim 120/C18/5 μm particle size/4.6 mm internal diameter/150 mm length) at room temperature. Solvent A (acidified water; 0.05 M) and solvent B (methanol) were used for the elution of the compound with a flow rate of 1 mL min^−1^. ABA detection was done at the absorbance of 254 nm.

Total SA content was assayed as explained by Pellegrini [51]. First, 150 mg of frozen leaf tissue was powdered with methanol (1 mL; 90% *v*/*v*) followed by vertexing, sonication (3 min), and centrifugation (10,000× *g*/10 min/room temperature) to obtain supernatant. Methanol (100% *v*/*v*; 0.5 mL) was used for re-extraction from the pellet. After sonication, the pellet was centrifuged, as explained above. Total supernatant was evaporated (40 °C/vacuum), residues were resuspended in TCA (0.25 mL; 5% *w*/*v*), and partitioning was done by adding ethyl acetate/cyclohexane (1:1 *v*/*v*; 0.8 mL). Free-SA content from the upper phase was concentrated under a vacuum at 40 °C. Conjugated SA in a lower phase was added with HCl (8 M; 0.3 mL) for hydrolysis, and incubation was done at 80 °C for 60 min. Total combined SA was dissolved in the mobile phase and methanol. Quantification of SA was done by taking absorbances at 305 nm and 407 nm with the flow rate (0.8 mL/min).

JA was assayed as explained by Pellegrini [51]. First, 150 mg of frozen leaf tissue was homogenized with ethyl acetate (1 mL) and incubated overnight (4 °C). The supernatant was harvested by centrifugation (10,000× *g*/10 min/4 °C) and mixed with acidified water (0.2% *v*/*v*), and the separated aqueous phase was analyzed with HPLC. JA was quantified by taking absorbance at 210 nm with the flow rate of 1 mL min^−1^.

### 2.6. Molecular Analysis for the Response of Disease-Resistance Genes

***Gene Expression Quantificationby RT-qPCR in Luffa.*** Gene expression analysis was done according to Rauf [52]. Total RNA was extracted from 150 mg of frozen leaf tissue by using the MiniBEST Plant RNA Extraction Kit (TaKaRa, Dalian, China). The integrity of RNA samples (260/280 nm absorbance ratio of 1.8–2.0) was evaluated by agarose gel (1%) electrophoresis. RevertAid First Strand cDNA Synthesifchens Kit by Invitrogen (Karlsruhe, Germany) was used to prepare cDNA from DNAase treated-RNA (2 μg). Gene expression analysis was done by using primers designed through primer 3.0 and the Primer-BLAST tool. Primer sequences are given in Table 1.

*Elongation factor 1α* (*EF-1α*) (MN548044) was used as a housekeeping gene (internal control), previously reported by Chen [53]. ABI PRISM 7900HT sequence detection system (Applied Biosystems Applera, Darmstadt, Germany) was used for gene amplification and real-time detection. SYBR Green (Applied Biosystems Applera, Darmstadt, Germany) master mix was used for gene amplification and detection of copy numbers. RT-qPCR for gene expression analysis was performed in triplicate.

***Gene Accession Numbers*.** Sequence data from this article can be found in the GenBank/EMBL databases under the following accession numbers: *JF414553.1* (*Cytochrome c oxidase subunit II*)*; JN039096.1* (*Translation elongation factor 1-like*)*; MW407164.1* (*RNA polymerase II*)*; KP641615.1* (*Chitinase*)*; MG702349.1* (*Endoglucanase*)*; XM_014103795.1* (*Subtilisin like protease*)*; NW_014013747.1* (*cellulase/glycoside hydrolase*)*; XM_014100723.1* (*NRPS dioxopiperazine synthetase; GliP*)*; FJ442590.1* (*Actin*)*; MG601052.1* (*Chitinase*)*; MG702349.1* (*Endoglucanase*)*; KC876057.1* (*subtilisin-like serine protease*)*; NW_020209251.1* (*cellulase/glycoside hydrolase*)*; XM_024916014.1* (*Squalene synthase*)*; FJ442452.1* (*Actin*)*; MN548044* (*Elongation factor 1α; EF-1α*)*; KP341758.1* (*Phenylalanine ammonia-lyase*)*; KM506755.1* (*Peroxidase*)*; KR819890.1* (*polyphenol oxidase*)*; KR184674.1* (*catalase*)*; KX092448.1* (*superoxide dismutase 3*)*; KX092439.1* (*L-Ascorbate peroxidase*)*; KX092434.1* (*Peroxidase 5*)*; MF678593.1* (*Gibberellin 2-beta-dioxygenase 8-like protein*)*; MF678591.1* (*ethylene-responsive transcription factor*)*; KX092444.1* (*Lipoxygenase; LOX*)*; MK649987.1* (*Zeaxanthin epoxidase; ZEP*)*; KX092441.1* (*9-cis-Epoxycarotenoid dioxygenase; NCED2*)*; MF678592.1* (*Abscisic acid receptor*)*; LC177373.1* (*Major latex-like protein*)*; JN230655.1* (*NBS-LRR resistance protein gene*)*; KX092450.1* (*Phytoene synthase; PSY*)*; AB033334.1* (*LcCAS1 mRNA for cycloartenol synthase*)*; and KR349345.1* (*Polyubiquitin; Ubi*).

### 2.7. Statistical Analysis

Three independent biological replicates with at least seven technical replicates were used for quantitative tests and measurements. Data analysis was done by using analysis of variance (ANOVA) and DMRT (Duncan Multiple Range Test) via SPSS-20 (SPSS Inch., Chicago, IL, USA) at *p* < 0.05.

## 3. Results

### 3.1. Isolation and Screening of Endophytic Fungal Isolates Based on Growth-Promoting Traits

**Figure 1:**
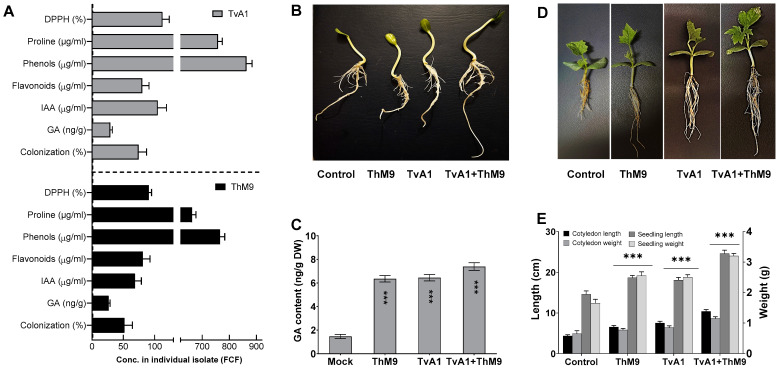
Characterization of ThM9 and TvA1 fungal isolates. (**A**) DPPH (%), proline, phenols, flavonoid content, IAA, GA, and root colonization. (**B**) Phenotypic analysis showing a comparison of ThM9 and TvA1 co-inoculation bioassay for germination and growth potential. (**C**) GA content in luffa seedling upon ThM9 and TvA1 co-inoculation bioassay. (**D**) Phenotypic analysis showing the comparison of ThM9 and TvA1 co-inoculation bioassay for plantlets of luffa. (**E**) Growth kinetics of luffa seedlings upon ThM9 and TvA1 co-inoculation bioassay. Quantitative data represent the means ± SD (n = 12) of three independent experiments. The asterisks indicate represents a significant difference between treated samples compared to control (*** *p* ≤ 0.005). FCF, fungal culture filtrate; DW, dry weight.

Initially, M9 and A1 were isolated from the host plant (*C. indica*). M9 and A1 were grown in Czapek media, and culture filtrate (CF) of M9 and A1 was quantified with a highly detectable amount of IAA, i.e., 53 ± 1.2 μg·mL^−1^ and 105 ± 1.3 μg·mL^−1^, respectively. GA content was 28 ± 0.5 μg·mL^−1^ and 35 ± 0.7 μg·mL^−1^, respectively. Both isolates ably synthesized a higher content of proline, i.e., 665 μg·mL^−1^ and 760 μg·mL^−1^; phenols, i.e., 764 μg·mL^−1^ and 876 μg·mL^−1^; and flavonoids, i.e., 86 μg·mL^−1^ and 79 μg·mL^−1^, respectively. Culture filtrates also exhibited strong DPPH free radical scavenging activity, i.e., 92 and 113%, respectively (Figure 1A).

### 3.2. Molecular Identification of Fungal Endophytes (M9 and A1) and Fungal Pathogen (DM1)

Molecular identification of M9 and A1 isolates was done by phylogenetic analysis. To this end, the *ITS1-5.8S-ITS2* region of *18S rDNA* gene sequences was subjected to NCBI database through nucleotide BLAST analysis (http://www.ncbi.nlm.nih.gov/, accessed on 22 April 2022). Through MEGA X software, phylogenetic analysis was performed to construct phylogenetic trees with the bootstrap replications of 1 K. Based on morphology and phylogenetic relationship, M9 and A1 were identified as members of *T. harzianum* and *T. virens* and putatively referred to as ThM9 and TvA1, respectively (Supplemental Figure S1). Further confirmation for species of the sequenced isolates was performed using species-specific primers. *Translation elongation factor 1-like* (*tef1*) gene-specific primers were used for *T. virens* genomic DNA that amplified ~573 bp fragment and *RNA polymerase II* (*RPB2*) gene-specific primers were used for *T. harzianum* genomic DNA that amplified~411 bp fragment. Purified fragments of *tef1* and *RPB2* were sequenced to confirm the species of ThM9 and TvA1. Sequence comparison was performed through BLAST search analysis followed by phylogenetic analysis. Based on the phylogenetic relationship, ThM9 and TvA1 were confirmed as *T. harzianum* and *T. virens* (Supplemental Figure S1).

### 3.3. Compatibility Test for ThM9 and TvA1

ThM9 and TvA1 isolates did not overgrow and restricted the growth of mycelium and sporulation for up to 8 days of incubation (Supplemental Figure S1), indicating that their compatibility with each other thus can be used in combination for potentially enhanced efficiency for plant growth.

### 3.4. Effect of ThM9 and TvA1 Endophytes on Growth Traits of Luffa under Laboratory Conditions

Compared to the control, the inoculation of ThM9 and TvA1 alone enhanced the growth traits of luffa plants, while the compatible combination of both ThM9 and TvA1 consortia further supported the growth significantly (*p* < 0.05) in terms of cotyledon and seedling length and weight (Figure 1). The effect of a symbiotic association of ThM9 and TvA1 alone and in combination with host-plant hormone GA was also evaluated. Results showed significantly (*p* < 0.05) higher GA content upon ThM9 and TvA1 inoculation compared to control. Further increase (7.6 ± 0.4 ng g^−1^ dry weight) in GA content was found upon consortial inoculation of ThM9 and TvA1 in luffa plants compared to individual inoculation and control plants (Figure 1).

### 3.5. Effect of ThM9 and TvA1 Endophytes on Seed Germination and Seedling Vigor of Luffa

All inoculations significantly enhanced seed germination and seedling vigor in comparison to control, with a variable rate of enhancement depending on the specific inoculation (ThM9, TvA1, and ThM9 + TvA1). Further, 88, 89, and 95% germination was observed upon ThM9, TvA1, and ThM9 + TvA1 inoculations, respectively, as against 84% in the control treatment. ThM9 + TvA1 exhibited significantly highest seedling vigor index (1983) among all the inoculations (ThM9, 1896 and TvA1,1895) and compared to control (1865) (Table 2).

### 3.6. Screening of ThM9 and TvA1 Endophytes for Antagonistic Response against Luffa DM

The effectiveness of ThM9 and TvA1 endophytic fungi against luffa DM pathogen (*P. cubensis*) was tested and evaluated using heavily infected, detached whole luffa leaves.

Mass production dominance of ThM9 and TvA1 endophytic fungi was evaluated in time-course manner by co-inoculating the detached whole luffa leaves heavily infected with the pathogen in 100 mL of Czapek culture broth media inoculated with 10 µL (1 × 10^8^ CFU/mL^−1^) of the conidial suspension of ThM9 and TvA1 endophytic fungi. Conidial suspension of each ThM9 and TvA1 endophytic fungi was found up to 1 × 10^8^ CFU/mL^−1^ using a hemocytometer even after 12 h.

Heavily infected leaves were sprayed with autoclaved Triton X-100 (0.01%) in ddH_2_O for collection of the conidia. The conidial suspension was centrifuged (10,000 rpm/10 min) and washed using the same solution as described by Chen [54].

Inhibition efficacy of endophytic fungi against the *P. cubensis* sporangia-releasing inhibition ratio (%) was evaluated from the co-inoculated assay. Sporangia-releasing inhibition assay revealed the efficient release of sporangia in the control culture after incubation (6 h), with the sporangia releasing ratio being 89.3%. ThM9- and TvA1-inoculated cultures showed obvious sporangia-releasing ratios of 79.1 and 81.4, respectively. Sporangia-releasing ratio (%) for ThM9 and TvA1 co-inoculation (95.1%) was tested to be more effective than that of ThM9 and TvA1 alone (Table 3).

### 3.7. The Predominance of Plant-Disease-Resistance Traits of Endophytes

**Figure 2:**
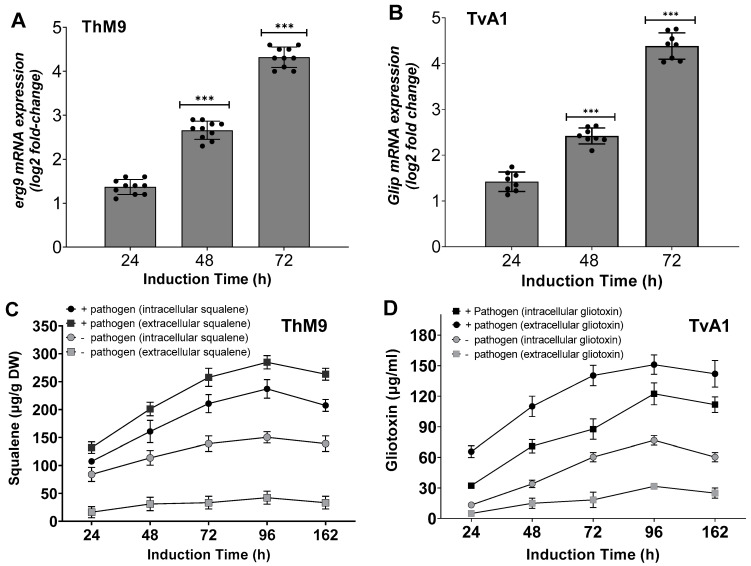
Induction of squalene and gliotoxin biosynthesis genes and metabolic level in ThM9 and TvA1 fungal isolates. (**A**) Expression of squalene biosynthesis enzymatic gene *SQS1/ERG9* (*squalene synthase*). (**B**) Expression of gliotoxin biosynthesis core enzymatic gene *GliP* (*NRPS dioxopiperazine synthetase*). (**C**) Squalene level in ThM9 and (**D**) gliotoxin level in TvA1 upon confrontation with *P. cubensis*. Quantitative data represent the means ± SD (n = 12) of three independent experiments. The asterisks indicate represents a significant difference between treated samples compared to control (*** *p* ≤ 0.005). DW, dry weight.

ThM9 and TvA1 endophytic fungal isolates showed functional traits well-known for induction of disease resistance responses. Induction of plant growth-promoting antifungal-bioactive compounds of ThM9 and TvA1 was achieved by setting up an infected leaf-dip culture assay for endophyte-pathogen confrontation. Culture filtrate exhibited a significantly (*p* < 0.05) higher squalene level produced (305-µg/g dry weight) in TvA1 culture upon confrontation with *P. cubensis* using infected leaf-dip culture assay compared to the control culture (153 µg/g dry weight). The transcript abundance of squalene biosynthesis enzymatic gene *SQS1/ERG9* was also induced in ThM9 upon confrontation with *P. cubensis*. Evaluation of the transcript abundance was performed by RT-qPCR analysis, and the data revealed an abundance of *SQS1/ERG9* transcript in ThM9 biomass of culture. Over time, the gliotoxin level increased (133-µg/mL) in *ThM9* culture upon confrontation with *P. cubensis* using infected leaf-dipped culture compared to the control culture that produced 81 µg/mL gliotoxin (Figure 2). *GliP* transcript (encoding the key gene for gliotoxin biosynthetic bimodular non-ribosomal peptide synthetase) showed abundance in TvA1 culture upon confrontation with *P. cubensis* using infected leaf-dipped culture compared to the control culture (Figure 2). These observations showed the high potential of ThM9 and TvA1 endophytes for producing plant-growth-promoting, antifungal-bioactive compounds over the time of endophyte–pathogen confrontation.

### 3.8. Induction of Defense-Related Hydrolytic Enzymes of ThM9 and TvA1

ThM9 and TvA1 isolates produced Chitinase, β-13-Endoglucanase, Subtilisin-like protease, and Glycoside hydrolase enzymes with maximum enzyme-specific activities. Evaluation for induction in gene expression of each respective enzyme was assessed by using endophytic cultures treated with *P. cubensis*-infected leaves of luffa in leaf-dip culture assay.

TvA1 culture filtrate exhibited significantly (*p* < 0.05) higher enzyme-specific activity after 36 h of induction for Chitinase (4.2 E Unit/mg protein), β-13-Endoglucanase (4.6 E Unit/mg protein), Subtilisin-like protease (4.7 E Unit/mg protein), and Glycoside hydrolase (4.1 E Unit/mg protein).

ThM9 culture filtrate exhibited significantly (*p* < 0.05) higher enzyme-specific activity for Chitinase (4.3E Unit/mg protein), β-13-Endoglucanase (4.4 E Unit/mg protein), Subtilisin-like protease (4.7 E Unit/mg protein), and Glycoside hydrolase (4.9 E Unit/mg protein) after 36 h of induction.

Similarly, TvA1 and ThM9 culture filtrates showed significantly (*p* < 0.05) higher gene expression for *Chitinase, β-13-Endoglucanase, Subtilisin-like protease*, and *Glycoside hydrolase* enzymes, even after 24 and 36 h of induction with *P. cubensis*-infected leaves of luffa in leaf-dip culture assay.

A significant (*p* < 0.005) gene expression induction in TvA1 culture for *Chitinase* was found up to 4.1-fold, *β-13-Endoglucanase* up to 4.7-fold, *Subtilisin-like protease* up to 4.4-fold, and *glycoside hydrolase* up to 4.2-fold after 36 h of induction.

**Figure 3:**
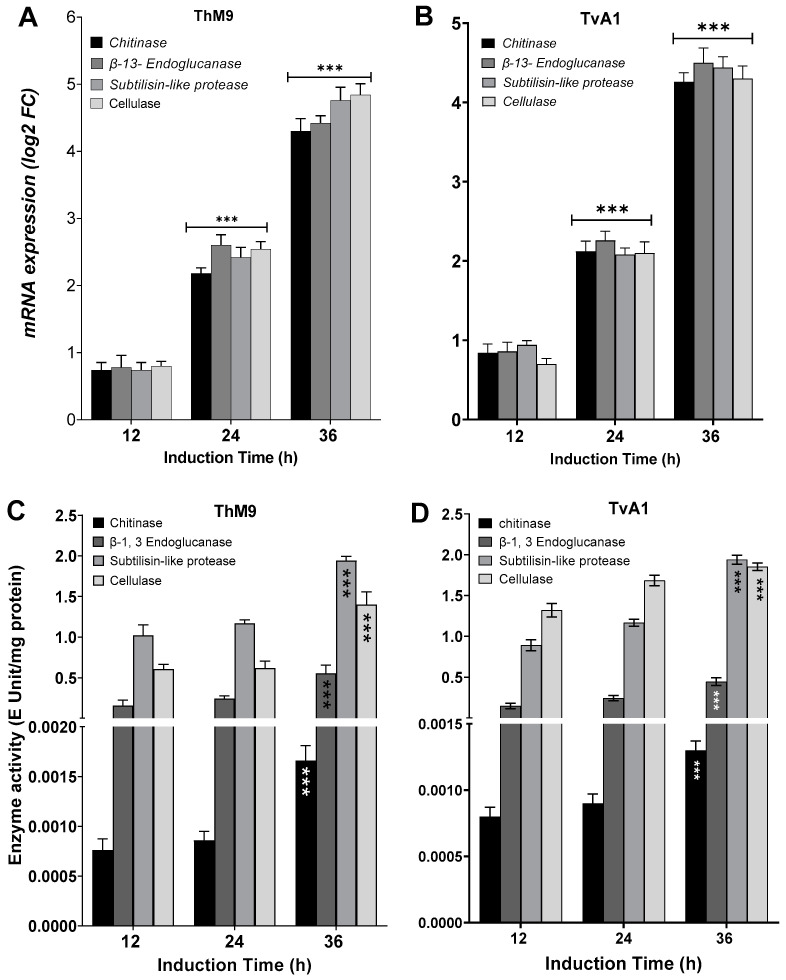
Induction of hydrolytic enzymes in ThM9 and TvA1 fungal isolates. (**A**) Expression of *Chitinase, β-13-Endoglucanase, Subtilisin-like protease*, and *glycoside hydrolase* enzymatic genes in ThM9. (**B**) Expression of *Chitinase, β-13-Endoglucanase, Subtilisin-like protease*, and *glycoside hydrolase* enzymatic genes in TvA1. (**C**) *Chitinase, β-13-Endoglucanase, Subtilisin-like protease*, and *glycoside hydrolase* enzymatic activities in ThM9. (**D**) *Chitinase, β-1,3-Endoglucanase, Subtilisin-like protease*, and *glycoside hydrolase* enzymatic activities in TvA1. Quantitative data represent the means ± SD (n = 12) of three independent experiments. The asterisks indicate represents a significant difference between treated samples compared to control (*** *p* ≤ 0.005).

Gene expression induction in ThM9 culture for *Chitinase* was significant (*p* < 0.005) and found to be up to 4.2-fold, *β-13-Endoglucanase* up to 4.4-fold, *Subtilisin-like protease* up to 5.0-fold, and *Glycoside hydrolase* up to 4.7-fold after 36 h of induction (Figure 3).

### 3.9. Effect of ThM9 and TvA1 Isolates on DM Disease Severity in Luffa

Preliminary effect of the fungal isolates (ThM9 and TvA1) and pathogen (*P. cubensis*) co-inoculation in luffa was investigated by using leaf disc and detached whole leaf bioassay, which permitted assessment of disease severity on luffa leaf discs in terms of disease severity index (%), electrolyte leakage (%), chlorophyll content, and stomatal conductance and transpirational activities for plant growth and survival. Quantitative data represented a significant (*p* < 0.005) reduction in chlorophyll content (9.0 SPAD value), and an increase in electrolyte leakage (33%) and disease severity index (>95%) was found in pathogen (*P. cubensis*)-inoculated luffa leaf discs compared to the control. The leaf discs inoculated with endophytic fungal isolates (ThM9 and TvA1) showed better responses against DM pathogen compared to the control. Significantly (*p* < 0.005) higher chlorophyll content retention, lower electrolyte leakage, and lesser disease severity were found in leaf discs inoculated with endophytic fungal isolates (ThM9 and TvA1) compared to control.

**Figure 4:**
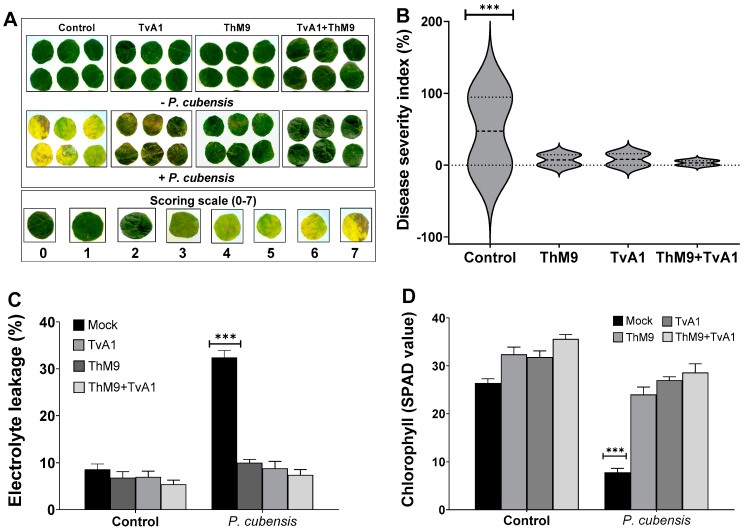
Preliminary effect of the fungal isolates (ThM9 and TvA1) and pathogen (*P. cubensis*) co-inoculation in luffa leaf disc bioassay. (**A**) Visualization of disease severity on luffa leaf discs upon co-inoculation with fungal endophytes and pathogen. (**B**) Disease severity index (%). (**C**) Electrolyte leakage (%). (**D**) Chlorophyll content. Quantitative data represent the means ± SD (n = 12) of three independent experiments. The asterisks indicate represents a significant difference between treated samples compared to control (*** *p* ≤ 0.005).

Quantitative data also showed a significant (*p* < 0.005) retention in chlorophyll content (28 SPAD value), reduction in electrolyte leakage (8%), and reduction in disease severity index (6%) in the leaf discs co-inoculated with fungal isolates (ThM9 and TvA1) and pathogen (*P. cubensis*) compared to the control (Figure 4).

**Figure 5:**
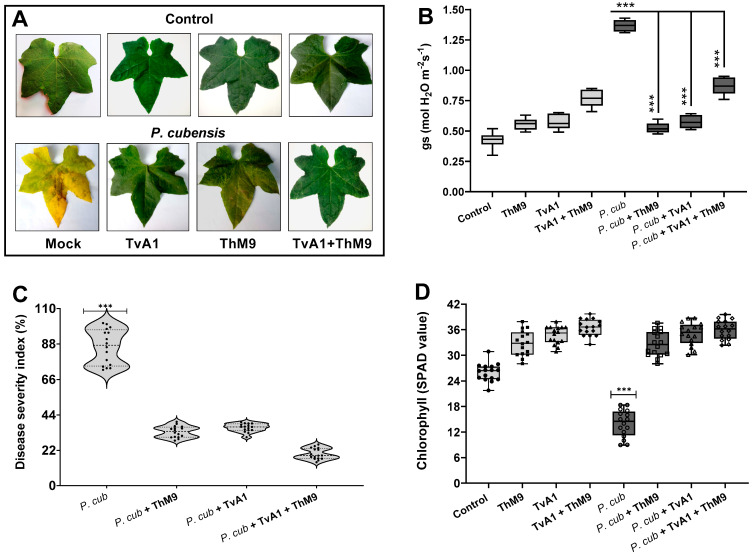
Effect of the fungal isolates (ThM9 and TvA1) and pathogen (*P. cubensis*) co-inoculation in luffa detached whole leaf bioassay. (**A**) Visualization of disease severity on luffa detached the whole leaf upon co-inoculation with fungal endophytes and pathogen. (**B**) Transpiration rate. (**C**) Disease severity index (%). (**D**) Chlorophyll content. Quantitative data represent the means ± SD (n = 12) of three independent experiments. The asterisks indicate represents a significant difference between treated samples compared to control (*** *p* ≤ 0.005).

Moreover, the effect of the co-inoculation of fungal isolates (ThM9 and TvA1) and pathogen (*P. cubensis*) in luffa detached whole leaf bioassay also showed a significant (*p* < 0.005) retention in chlorophyll content (34 SPAD value) and reduction in disease severity index (6%) and stomatal conductance (0.76 mol H_2_O m^−2^s^−1^) compared to the control (Figure 5).

### 3.10. Effect of ThM9 and TvA1 Isolates on Oxidative Status of Infected Luffa Leaf

**Figure 6:**
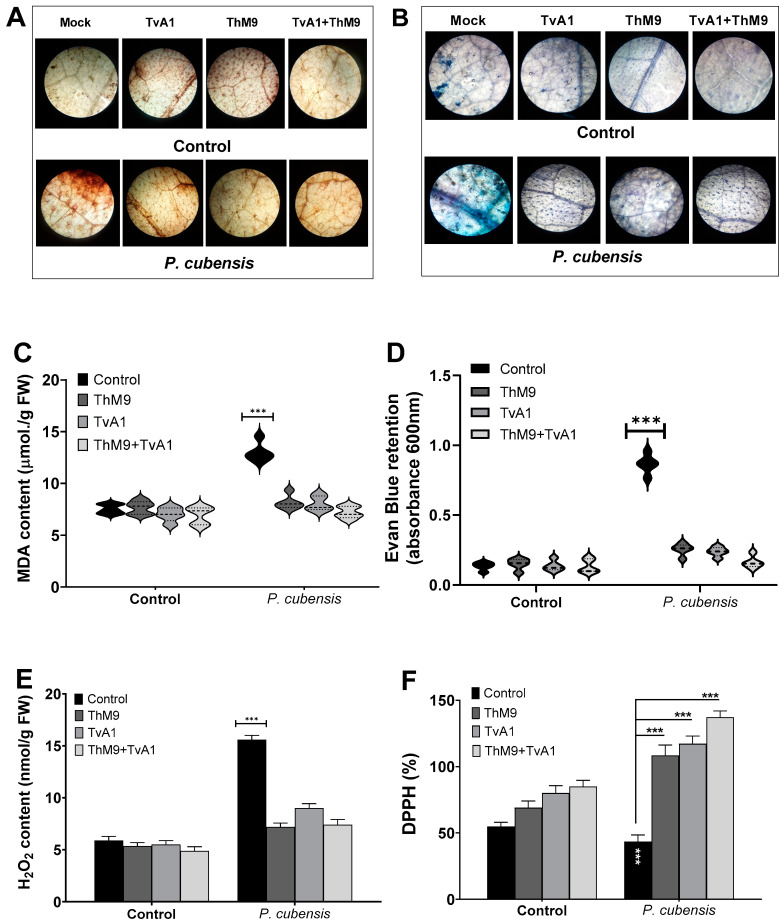
Effect of the fungal isolates (ThM9 and TvA1) and pathogen (*P. cubensis*) co-inoculation on oxidative status and antioxidant potential in luffa leaf. (**A**) DAB staining. (**B**) Evan blue staining. (**C**) MDA content. (**D**) Retention of Evan blue. (**E**) H_2_O_2_ content. (**F**) DPPH (%). Quantitative data represent the means ± SD (n = 12) of three independent experiments. The asterisks indicate a significant difference between treated samples compared to control (*** *p* ≤ 0.005). FW, fresh weight.

ThM9 and TvA1 isolates effectively reduced the DAB and Evan blue retention, MDA, and H_2_O_2_ content (Figure 6) and increased the DPPH (%) in pathogen-inoculated leaves of luffa, thus protecting membrane integrity upon infection. Therefore, the disease index calculated for luffa was significantly reduced with low Evan blue retention in ThM9 and TvA1-treated plants.

**Figure 7:**
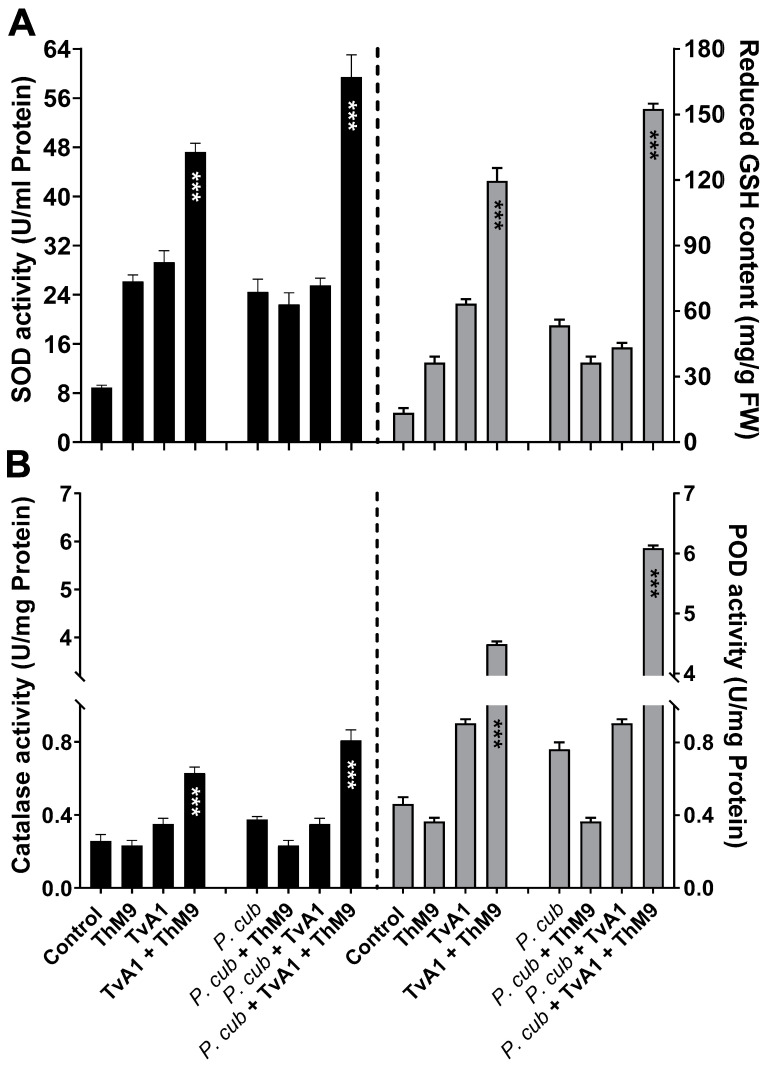
Effect of the fungal isolates (ThM9 and TvA1) and pathogen (*P. cubensis*) co-inoculation on antioxidant enzyme activities in luffa leaf. (**A**) SOD (left axis), GSH content (right axis). (**B**) CAT (left axis), POD (right axis). Quantitative data represent the means ± SD (n = 12) of three independent experiments. The asterisks indicate represents a significant difference between treated samples compared to control (*** *p* ≤ 0.005). FW, fresh weight.

Antioxidant enzyme activities were also modulated upon ThM9 and TvA1 supplementation in luffa plants infected with a pathogen (*P. cubensis*). The DM infection significantly (*p* < 0.005) increased SOD activity (2.6-fold) and GSH content (5.7-fold) in comparison to control, while luffa plants pre-inoculated with ThM9 and TvA1 and infected with a pathogen (*P. cubensis*) resulted in a further increase in the SOD activity (7.8-fold) and GSH content (15-fold) in comparison to control plants (Figure 7A).

Similarly, POD and CAT activity assessment showed a significant (*p* < 0.05) elevation in CAT (1.37-fold) and POD activity (7.7-fold) in luffa plants infected with a pathogen (*P. cubensis*) compared to control, while luffa plants pre-inoculated with ThM9 and TvA1 and infected with a pathogen (*P. cubensis*) showed further increase in POD (15-fold) and CAT activity (3-fold) compared to other treatments (Figure 7B).

### 3.11. Effect of ThM9 and TvA1 Isolates on Phytohormonal Content of Infected Luffa Plants

At the basal level, ACC, JA, ABA, and SA were detectible in all the treatments of seedlings (with or without pathogen inoculation); however, in endophyte pre-inoculated (ThM9, TvA1, and ThM9 + TvA1) seedlings, the level of ACC, JA, ABA, and SA was significantly higher than control seedlings.

**Figure 8:**
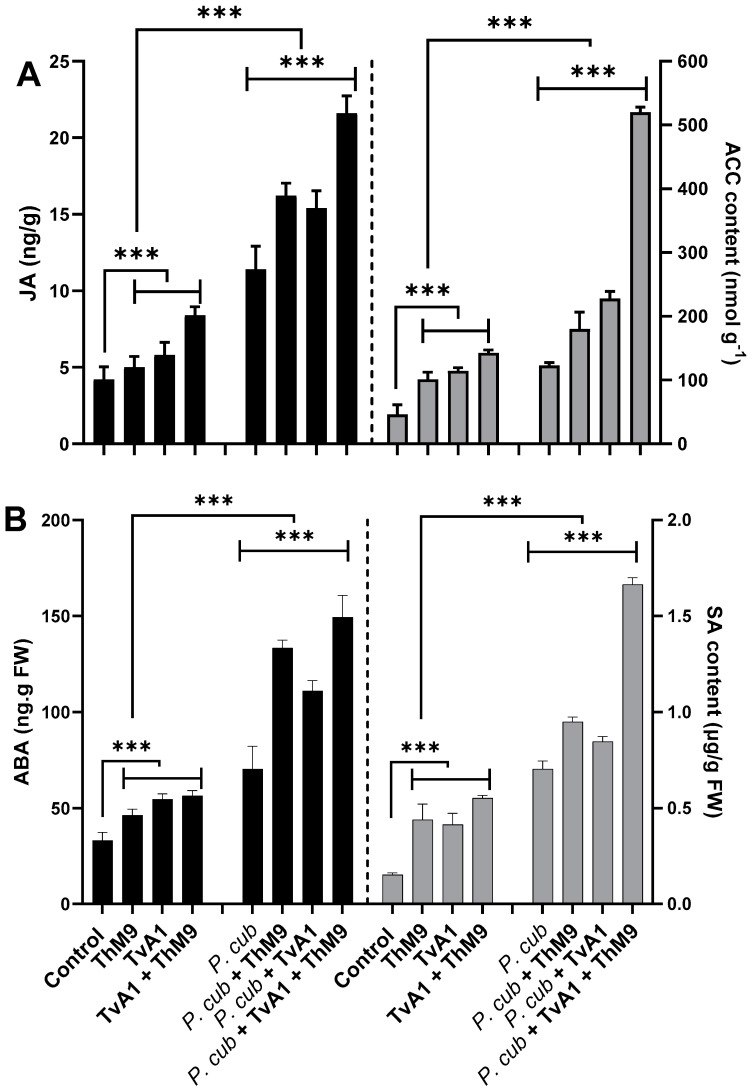
Effect of the fungal isolates (ThM9 and TvA1) and pathogen (*P. cubensis*) co-inoculation on phytohormonal content in luffa leaf (**A**) JA (left axis), ACC contents (right axis). (**B**) ABA (left axis), SA (right axis). Quantitative data represent the means ± SD (n = 12) of three independent experiments. The asterisks indicate a significant difference between treated samples compared to control (*** *p* ≤ 0.005). FW, fresh weight.

***ACC content*.** ACC content was quantified to analyze the regulation of the physiological scenario of the luffa plants under normal and diseased conditions with and without ThM9 and TvA1 pre-inoculation (Figure 8), and the results showed a significant (*p* < 0.05) accumulation in ACC content of pathogen-non-infected, endophyte-pre-inoculated seedlings up to 2-fold for ThM9 and 2.1-fold for TvA1 compared to control seedlings (non-infected and non-inoculated). Consortial inoculation of ThM9 and TvA1 further induced the accumulation of ACC content (3-fold) compared to control seedlings (non-infected and non-inoculated). Pathogen-inoculated seedlings also showed a significant (*p* < 0.05) accumulation in ACC content (2.8-fold) in comparison to control seedlings (non-infected and non-inoculated), while, following the pathogen inoculation, ACC content in endophyte-pre-inoculated seedlings with ThM9, TvA1, and ThM9 + TvA1 was further increased (3.8-fold, 4.2-fold, and 10.2-fold, respectively) compared to control seedlings (non-infected and non-inoculated) (Figure 8A).

***JA content*.** The results showed a significant (*p* < 0.005) abundance in JA content of non-infected, endophyte-pre-inoculated seedlings (1.2-fold) for ThM9 and 1.4-fold for TvA1 compared to control seedlings (non-infected and non-inoculated).

Consortial inoculation of ThM9 and TvA1 further induced the accumulation of JA content (2-fold) compared to control seedlings (non-infected and non-inoculated).

JA content was quantified, and results showed that there was a significant (*p* < 0.005) increase in JA content of pathogen-inoculated seedlings without endophytic inoculation (3-fold) compared to control seedlings (non-infected and non-inoculated).

Following the pathogen inoculation, JA content in endophyte-pre-inoculated seedlings with ThM9, TvA1, and ThM9 + TvA1 was further increased (4.1-fold, 3.75-fold, and 5.75-fold, respectively) compared to control seedlings (non-infected and non-inoculated) (Figure 8A).

***SA content*.** The results revealed a significant (*p* < 0.005) elevation in SA content of non-infected, endophyte-pre-inoculated (ThM9 and TvA1) seedlings (4-fold for ThM9 and 3.8-fold for TvA1) compared to control seedlings (non-infected and non-inoculated).

Consortial inoculation of ThM9 and TvA1 further induced the accumulation of SA content (5-fold) in comparison to the control seedlings (non-infected and non-inoculated).

SA content was quantified, and results showed that there was a significant (*p* < 0.005) increase in SA content (7.5-fold) of pathogen-inoculated seedlings without endophytic inoculation compared to control seedlings (non-infected and non-inoculated).

Following the pathogen inoculation, SA content in endophyte-pre-inoculated seedlings with ThM9, TvA1, and ThM9 + TvA1 was further increased (9.5-fold, 8-fold, and 16-fold, respectively) compared to the control seedlings (non-infected and non-inoculated) (Figure 8B).

***ABA content*.** The results exhibited a significant (*p* < 0.005) accumulation in ABA content of non-infected, endophyte-pre-inoculated (ThM9 and TvA1) seedlings (1.2-fold for ThM9 and 1.6-fold for TvA1) compared to control seedlings (non-infected and non-inoculated).

Consortial inoculation of ThM9 and TvA1 further induced the accumulation of ABA content (1.8-fold) compared to control seedlings (non-infected and non-inoculated).

ABA content was quantified, and results showed that there was a significant (*p* < 0.005) increase in ABA content of pathogen-inoculated seedlings without endophytic inoculation (1.9-fold) compared to control seedlings (non-infected and non-inoculated).

Following the pathogen inoculation, ABA content in endophyte-pre-inoculated seedlings with ThM9, TvA1, and ThM9 + TvA1 was further increased (3.5-fold, 2.75-fold, and 3.75-fold, respectively) compared to control seedlings (non-infected and non-inoculated) (Figure 8B).

### 3.12. Effect of ThM9 and TvA1 isolates on gene expression of infected luffa plants

**Figure 9:**
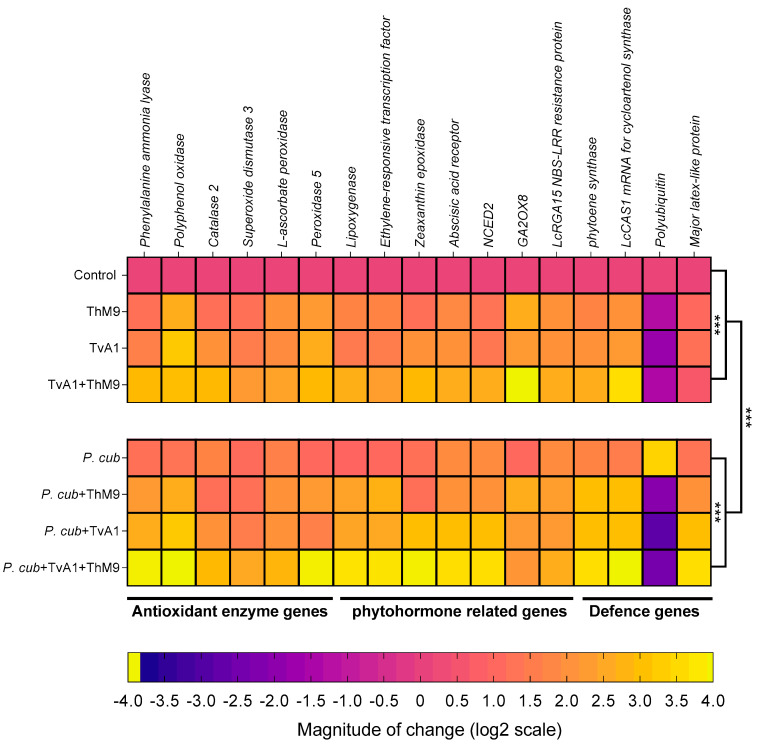
Expression profiling of antioxidant enzymatic genes, phytohormonal biosynthesis, and signaling genes and defense-related genes in luffa leaf upon co-inoculation of the fungal isolates (ThM9 and TvA1) and pathogen (*P. cubensis*) by RT-qPCR. Quantitative data represent the means ± SD (n = 12) of three independent experiments. The asterisks indicate a significant difference between treated samples compared to control (*** *p* ≤ 0.005).

The transcript abundance of antioxidant and defense-related enzymatic marker genes (*PPO, PAL, CAT2, SOD3, L-ascorbate peroxidase, POD5*), phytohormonal genes (*LOX, ERF, ZEP, abscisic acid receptor, NCED2, GA2OX8, NBS-LRR resistance protein*), and defense-proteins-related genes (*PSY, LcCAS1, polyubiquitin, MLP3*) were evaluated as shown in Figure 9.

***Phenylalanine ammonia-lyase* (*PAL*).***PAL* gene encoding a phenol and SA biosynthesis key enzyme Phenylalanine ammonia-lyase was induced in ThM9- and TvA1-pre-inoculated seedlings 1.1-fold and 1.5-fold, respectively, compared to control seedlings (non-infected and non-inoculated), while further induction was noticed in the expression of *Phenylalanine ammonia-lyase* (2.8-fold) in luffa seedlings pre-treated with ThM9 and TvA1 both compared to control plants. Pathogen-inoculated seedlings without endophytic pre-treatment showed an increased gene expression up to 1.1-fold compared to control plants, while seedlings inoculated with the pathogen and pre-inoculated with a combination of ThM9 and TvA1 endophytes showed the highest increase in *PAL* gene expression up to 3.8-fold compared to control plants (Figure 9).

***Polyphenol oxidase* (*PPO*).***PPO* gene encoding polyphenol biosynthesis enzyme Polyphenol oxidase showed 2.5-fold and 3.1-fold increase in ThM9- and TvA1-pre-inoculated seedlings compared to control. Upon consortial pre-treatment with ThM9 and TvA1, a 2.9-fold increase was noticed for *PPO* gene expression compared to control.

Infected seedlings without endophytic pre-treatment showed an increased gene expression up to 1.2-fold compared to control plants, while seedlings inoculated with the pathogen and pre-inoculated with the combination of ThM9 and TvA1 endophytes showed the highest increase in *Polyphenol oxidase* gene expression up to 3.9-fold compared to control seedlings (Figure 9).

***Catalase 2* (*CAT2*).***CAT2* gene encoding ROS scavenger enzyme Catalase 2 showed 1.06-fold and 2.8-fold increase in ThM9- and TvA1-pre-inoculated seedlings compared to control. Upon consortial pre-inoculation with ThM9 and TvA1, a 2.8-fold increase was noticed for *Catalase 2* gene expression compared to control. Pathogen-inoculated seedlings without endophytic pre-treatment showed an increased gene expression up to 1.6-fold compared to control plants, while seedlings inoculated with the pathogen and pre-inoculated with a combination of ThM9 and TvA1 endophytes showed the highest increase in *CAT2* gene expression up to 2.8-fold compared to control seedlings (Figure 9).

***Superoxide dismutase 3* (*SOD3*).***SOD3* gene encoding ROS scavenger enzyme Superoxide dismutase 3 showed a 1.1-fold and 1.4-fold increase in ThM9- and TvA1-pre-inoculated seedlings compared to control. Upon consortial pre-treatment with ThM9 and TvA1, a 2.1-fold increase was noticed for *superoxide dismutase 3* gene expression compared to control.

Infected seedlings without endophytic pre-treatment showed an increased gene expression up to 1-fold compared to control plants, while seedlings inoculated with the pathogen and pre-inoculated with a combination of ThM9 and TvA1 endophytes showed the highest increase in *SOD3* gene expression up to 2.4-fold compared to control seedlings (Figure 9).

***L-ascorbate peroxidase* (*APX*).***APX* gene encoding ROS scavenger enzyme L-Ascorbate peroxidase showed a 1.9-fold and 1.8-fold increase in ThM9- and TvA1-pre-inoculated seedlings compared to control. Upon consortial pre-treatment with ThM9 and TvA1, a 2.3-fold increase was noticed for *L-Ascorbate peroxidase* gene expression compared to control.

Infected seedlings without endophytic pre-treatment showed an increased *L-Ascorbate peroxidase* gene expression (1.5-fold) compared to control plants, while seedlings inoculated with the pathogen and pre-inoculated with a combination of ThM9 and TvA1 endophytes showed the highest increase in *APX* gene expression up to 2.7-fold compared to control seedlings (Figure 9).

***Peroxidase 5* (*POD5*).***POD5* gene encoding ROS scavenger enzyme Peroxidase 5 showed 2.1-fold and 2.5-fold increase in ThM9- and TvA1-pre-inoculated seedlings compared to control. Upon consortial pre-treatment with ThM9 and TvA1, a 2.8-fold increase was noticed for *POD5* gene expression compared to control. Infected seedlings without endophytic re-treatment showed an increased gene expression up to 0.9-fold compared to control plants. Infected seedlings and pre-inoculated with a combination of ThM9 and TvA1 endophytes showed the highest increase in *POD5* gene expression up to 3.8-fold compared to control seedlings (Figure 9).

***Lipoxygenase* (*LOX*).***LOX* gene encoding a JA biosynthesis enzyme Lipoxygenase showed a 1.6-fold and 1.3-fold increase in ThM9 and TvA1 pre-inoculated seedlings compared to control. Upon consortial pre-treatment with ThM9 and TvA1, a 2.5-fold increase was noticed for *lipoxygenase* gene expression compared to control. Pathogen-inoculated seedlings without endophytic pre-treatment showed an increased gene expression up to 0.8-fold compared to control plants. Infected seedlings and pre-inoculated with a combination of ThM9 and TvA1 endophytes showed the highest increase in *LOX* gene expression (3.5-fold) compared to control seedlings (Figure 9).

***Ethylene-responsive transcription factor* (*ERF*).***ERF* gene encoding Ethylene-responsive transcription factor showed a 1.6-fold and 1.4-fold increase in ThM9- and TvA1-pre-inoculated seedlings compared to control. Upon consortial pre-treatment with ThM9 and TvA1, a 2.2-fold increase was noticed for *ERF* gene expression compared to control. Infected seedlings without endophytic pre-treatment showed an increased gene expression up to 0.9-fold compared to control plants, while infected seedlings and pre-inoculated with a combination of ThM9 and TvA1 endophytes showed the highest increase in *ERF* gene expression up to 3.6-fold compared to control seedlings (Figure 9).

***Zeaxanthin epoxidase* (*ZEP*).***ZEP* gene encoding an ABA biosynthesis enzyme Zeaxanthin epoxidase showed a 1.5-fold and 1.9-fold increase in ThM9- and TvA1-pre-inoculated seedlings compared to control. Upon consortial pre-treatment with ThM9 and TvA1, a 2.8-fold increase was noticed for *ZEP* gene expression compared to control. Pathogen-inoculated seedlings without endophytic pre-treatment showed an increased gene expression up to 1.1-fold compared to control plants, while infected seedlings and pre-inoculated with a combination of ThM9 and TvA1 endophytes showed the highest increase in *ZEP* gene expression up to 3.8-fold compared to control seedlings (Figure 9).

***Abscisic acid receptor*.***Abscisic acid receptor* gene encoding abscisic acid receptor showed a 1.7-fold and 1.9-fold increase in ThM9- and TvA1-pre-inoculated seedlings compared to control. Upon consortial pre-treatment with ThM9 and TvA1, a 2.5-fold increase was noticed for *Abscisic acid receptor* gene expression compared to control. Pathogen-inoculated seedlings without endophytic pre-treatment showed an increased gene expression up to 1.8-fold compared to control plants, while infected seedlings and pre-inoculated with a combination of ThM9 and TvA1 endophytes showed the highest increase in *Abscisic acid receptor* gene expression (3.5-fold) compared to control seedlings (Figure 9).

***9-cis-epoxycarotenoid dioxygenase*(*NCED2*).***NCED2* gene encoding 9-cis-epoxycarotenoid dioxygenase enzyme showed increased expression in ThM9- and TvA1-pre-inoculated seedlings (1.2-fold and 1.3-fold, respectively) compared to control. Upon consortial pre-treatment with ThM9 and TvA1, a 2.5-fold increase was noticed for *NCED2* gene expression compared to control. Pathogen-inoculated seedlings without endophytic pre-treatment showed an increased gene expression up to 1.8-fold compared to control plants, while infected seedlings and pre-inoculated with a combination of ThM9 and TvA1 endophytes showed the highest increase in *NCED2* gene expression (3.5-fold) compared to control seedlings (Figure 9).

***Gibberellin 2-beta-dioxygenase 8* (*GA2OX8*).** GA biosynthesis enzymatic gene *GA2OX8* showed 2.5-fold and 2.1-fold increase in ThM9- and TvA1-pre-inoculated seedlings compared to control. Upon consortial pre-treatment with ThM9 and TvA1, a 3.9-fold increase was noticed for *GA2OX8* gene expression compared to control.

Pathogen-inoculated seedlings without endophytic pre-treatment showed an increased gene expression up to 0.9-fold compared to control plants, while seedlings inoculated with the pathogen and pre-inoculated with a combination of ThM9 and TvA1 endophytes showed the highest increase in *GA2OX8* gene expression (2.0-fold) compared to control seedlings (Figure 9).

***NBS-LRR-resistance protein*.** Gene for NBS-LRR-resistance protein showed a 1.9-fold and 1.9-fold increase in ThM9- and TvA1-pre-inoculated seedlings compared to control. Upon consortial pre-treatment with ThM9 and TvA1, a 2.5-fold increase was noticed for *NBS-LRR-resistance protein* gene expression compared to control. Pathogen-inoculated seedlings without endophytic pre-treatment showed an increased gene expression up to 1.8-fold compared to control plants, while infected seedlings and pre-inoculated with a combination of ThM9 and TvA1 endophytes showed the highest increase in *NBS-LRR-resistance protein* gene expression (2.5-fold) compared to control seedlings (Figure 9).

***Phytoene synthase* (*PSY*).***PSY* gene encoding carotenoid biosynthesis Phytoene synthase enzyme showed a 1.6-fold and 1.9-fold increase in ThM9- and TvA1-pre-inoculated seedlings compared to control. Upon consortial pre-treatment with ThM9 and TvA1, a 2.5-fold increase was noticed for *PSY* gene expression compared to control. Pathogen-inoculated seedlings without endophytic pre-treatment showed an increased gene expression up to 1.6-fold compared to control plants, while infected seedlings and pre-inoculated with a combination of ThM9 and TvA1 endophytes showed the highest increase in *PSY* gene expression (3.5-fold) compared to control seedlings (Figure 9).

***Cycloartenol synthase* (*CAS1*).***CAS1* gene encoding Cycloartenol synthase enzyme involved in the biosynthesis of cycloartenol (an important plant steroid belonging to the triterpenoid of the sterol class) showed a 1.9-fold and 2.1-fold increase in ThM9- and TvA1-pre-inoculated seedlings compared to control. Upon consortial pre-treatment with ThM9 and TvA1, a 3.5-fold increase was noticed for *CAS1* gene expression compared to control. Infected seedlings without endophytic pre-treatment showed an increased gene expression up to 1.4-fold compared to control plants, while infected seedlings and pre-inoculated with a combination of ThM9 and TvA1 endophytes showed the highest increase in *CAS1* gene expression up to 3.9-fold compared to control seedlings (Figure 9).

***Polyubiquitin* (*Ubi*).***Polyubiquitin* gene showed a –1.4-fold and –1.9-fold decrease in ThM9- and TvA1-pre-inoculated seedlings respectively, compared to control. Upon consortial pre-treatment with ThM9 and TvA1, a –1.5-fold decrease was noticed for *Polyubiquitin* gene expression compared to control, while pathogen-inoculated seedlings without endophytic pre-treatment showed an increased gene expression up to 3.3-fold compared to control plants. Moreover, infected seedlings and pre-inoculated with a combination of ThM9 and TvA1 endophytes showed a decrease in *Polyubiquitin* gene expression (2.5-fold) compared to control seedlings (Figure 9).

***Major latex protein-like protein 43* (*MLP43*).***MLP43* gene encoding for Major latex protein-like protein 43 known for its functions as a positive regulator during abscisic acid responses and confers drought tolerance in *Arabidopsis. MLP43* gene expression showed up to 0.9- and 1.1-fold increase in ThM9 and TvA1 pre-inoculated seedlings, respectively, compared to control. Upon consortial pre-treatment with ThM9 and TvA1, a 0.5-fold increase was noticed for *MLP43* gene expression compared to control, while pathogen-inoculated seedlings without endophytic pre-treatment showed an increased gene expression up to 1.2-fold compared to control plants. Moreover, infected seedlings and pre-inoculated with a combination of ThM9 and TvA1 endophytes showed a decrease in *MLP43* gene expression (3.5-fold) in comparison to control (Figure 9).

## 4. Discussion

*Trichoderma* is a previously well-characterized endophytic fungus known to either secrete GAs or IAA, which improve plant growth and crop productivity. However, not so many have been characterized so far that produce defense-related SMs essential for the survival of plants against fungal pathogens to alleviate biotic stress. Increasing research on the identification and characterization of biocontrol agents (BCA) against fungal phytopathogens has explored the much more efficient role of *Trichoderma* (*T. harzianum, T. asperellum*, and *T. virens*) among the most suitable BCA against fungal phytopathogens, such as *B. cinerea, P. megakarya, S. sclerotium, P. capsici, S. fusca, P. citrophthora, P. erythroseptica, P. palmivora*, and *P. ultimum* as well as *P. cubensis* (a causative agent for DM disease in plants) [55].

In the current research, we analyzed the effectiveness of novel and previously unreported endophytic fungal isolates *T. harzianum* (ThM9) and *T. virens* (TvA1) as growth promoters for luffa plants. The current experimental setup also shed light on the fact that the consortial inoculation of ThM9 and TvA1 is the best combination of most compatible plant growth-promoting endophytes having the potential to positively immunomodulate the biotic stress tolerance in luffa and significantly ameliorate the adverse pathogenic effects by physiological, bio-chemical, and molecular-adaptative strategies. Under the current study, the ThM9 and TvA1 exhibited higher production of IAA, GA, proline, phenols, and flavonoids with high DPPH free radical scavenging potential and efficiently induced luffa seedling growth.

Moreover, growth-promoting phytohormone GA biosynthesis enzymatic gene *GA2OX8* showed increased expression and higher GA content in ThM9- and TvA1-pre-inoculated luffa seedlings compared to control, further indicating the growth-promoting effect of ThM9 and TvA1 fungal endophytes. Several *Trichoderma*, including *T. asperellum, T. afroharzianum, T. longibrachiatum, T. virens*, and *T. lixii*, are reported to enhance the photosynthetic potential of plants [56]. The synthesis of phytohormones is also enhanced in response to changing environmental conditions (biotic and abiotic stress), suggesting that they play a role in bridging the gap between changing environments and developmental adaptation. The many features of ET as a signaling molecule, for example, have shown substantial biochemical links between ET and growth control. ET biosynthesis is known to be triggered by heat, submergence, shade, high salt, exposure to heavy metals stress, water deficiency, and low nutrient availability [24,57]. *Trichoderma* is ubiquitous, and their supplementation in nutrient-deficient soil proved efficient in nutrient solubilization leading to soil quality enhancement [58] and *Trichoderma* strains also induce plant resistance to biotic stresses through systemic defense (SAR and ISR) and enhance plant growth with improved yield.

*Trichoderma* aggressiveness against phytopathogens is linked with the secretion of trichotoxins that promote plant growth and stimulate ISR in plants, equally effectively as that provided by chemical materials or fungicides. *P. cubensis* (an obligate biotrophic oomycete) is a DM-causative pathogen for cucurbit foliar disease that defoliates the crop and results in great economic losses. DM of luffa is the crucial biotic constraint to its production throughout Asia, and controlling its pathogen is a major challenge for luffa growers, as it influences the biochemical and molecular scenarios of plant growth and leads to physiological as well as morphological irregularities and deformities in various plants. In the current research, we found that application of luffa leaf disc tissue and whole detached leaf with ThM9 and TvA1 reduced the DM development and progression upon pathogen inoculation, indicating the positive role of ThM9 and TvA1 endophytes against DM pathogen attack on luffa.

*Trichoderma*-mediated modulation of the plant transcriptome, proteome, and metabolome has been extensively investigated; for example, *Trichoderma* has also been explored as an elicitor of wheat-plant resistance responses against *S. tritici* [59]. Moreover, transcriptomic changes have been observed to induce plant defense responses in wheat roots that interacted with *T. harzianum* [60]. Previous reports have also exposed mycoparasitic fungi, including *Trichoderma*, showing antagonistic activity against pathogens (*Verticillium, Rhizoctonia, Sclerotinia, Pythium, Fusarium*) by initiating the induction of systemic resistance (ISR) through complex adaptive mechanisms, including lytic enzymes secretion [61,62]. Previously, it was also made known that an efficient BCA exhibits more than one mechanism of action against phytopathogens, namely “synergism”, including (1) antibiosis that is concerned with the biosynthesis and secretion of chemicals that restrict the growth of pathogens [63]; (2) competition for nutrients and superficial growth: this mechanism is active against pathogen reproductive cycle by inhibiting conidial growth and germination through exogenous nutrient depletion, necessary for germination process; and (3) mycoparasitism that deals with the production and secretion of pathogen cell-wall-degrading enzymes such as chitinases (endochitinases, β-*N*-acetilhexosaminidases, and exochitinases), cellulases (exoglucanases, endoglucanases, and β-1-3-glucanases), and proteases [64]. *Trichoderma sp*. are well-reported for producing antifungal compounds and SMs against phytopathogenic fungi, namely Epipolythiodioxopiperazines (ETPs), such as gliotoxin, ergosterol derivatives, and precursors (squalene), and peptaibols, etc. [65]. Ergosterol has been known as one of the most important elicitors of plant defense responses by induction of an oxidative burst in plants [66]. Several lines of evidence support the role of JA signaling as well in the ergosterol-elicitation response [67]. Ergosterol elicitation is reported to ably regulate the defense-related genes upon pathogens’ attack [21].

The biosynthesis of sterols is tightly controlled at multiple levels. SQS1/Erg9 and Erg1 are key enzymes in the sterol-biosynthesis pathway [37]. SQS1/Erg9 encodes farnesyl-diphosphate farnesyl transferase (squalene synthase) and connects two farnesyl pyrophosphate moieties to form squalene (a key intermediate in the sterol biosynthesis). Squalene (cell membranes localized, hydrophobic neutral lipid, and polyunsaturated terpene) is a rate-limiting intermediate in the ergosterol biosynthetic pathway. Fluctuation in the level of squalene and ergosterol modulates the fluidity or rigidity of the fungal membranes [68]. Another group of antifungal SMs is gliotoxins (sulfur-containing mycotoxins) produced by several fungal species, e.g., *T. virens* (*Gliocladium virens*)*, A. fumigatus, P. obscurum*, and *Acremonium* sp. [23,69]. They have antifungal, antimicrobial, antiviral, and immunomodulating properties. Gliotoxin biosynthesis genes are present only in five species of *Trichoderma* (*T. virens*, *T. harzianum*, *T. afroharzianum*, *T. reesei*, and *T. longibrachiatum*). GliP (NRPS dioxopiperazine synthetase; a non-ribosomal peptide synthetase enzyme) is involved in the biosynthesis of cyclo-phenylalanyl-serine (first biosynthetic intermediate in the gliotoxin biosynthetic pathway), while *GliP* deletion resulted in complete loss of gliotoxin biosynthesis [70]. At the same time, in current research, we found that TvA1 and ThM9 isolates also exhibited induction of antifungal-bioactive compounds (squalene and gliotoxin, respectively) upon *P. cubensis* confrontation, along with the inducible gene expression for squalene (*SQS1/ERG9* gene) and gliotoxin (*GliP* gene) biosynthesis. These preliminary findings attracted the main focus of current research towards the exploitation of the ThM9 and TvA1 isolates as BCAs against *P. cubensis* pathogenic fungus of luffa for a DM cure. To our knowledge, there is no previous study on the exploitation of *Trichoderma* as BCA against DM of luffa and the underlying molecular mechanism governing the DM resistance. In general, endophytic fungi also enhance the defense mechanism more efficiently through antibiotic activity combined with lytic enzymes production [71]. reported that the cell walls disintegration of *B. cinerea* and *F. oxysporum* by lytic enzymes is enhanced through antibiotic penetration into the target hypha. The *T. virens* gene *veA* ortholog *vel1* encodes the VELVET protein and modulates the biosynthesis and the biocontrol activity induced by gliotoxin as well as other genes expressions participating in the SMs. Deletion of *veA* resulted in a reduction of gene expression of *GliZ* and *GliP*, and a reduction of protease activity essential for controlling fungal hydrolytic activity and *T. reesei vel1*—such as *lae1*—is also essential for cellulase and hemicellulase gene expression [72]. Cellulases from *Trichoderma* also induced the ISR in plants via ET or JA pathways [73]. Cellulase-like proteins *Thph2* and *Thph1* from *T. harzianum* triggered the ISR in maize leaves, while fungal deletion mutant lost the ability to activate maize immune response against *C. lunata* (*fungal pathogen*) [74]. Consistently, our study also revealed the higher cellulase enzyme activity exhibited by ThM9 and TvA1 fungal isolates upon confrontation with *P. cubensis* pathogen. Time-course activation of cellulase gene expression by ThM9 and TvA1 also revealed the co-relation of its gene-inducibility pattern to that of initiation of the mycoparasitic response of endophytic fungus. Moreover, the overaccumulation of ET and JA levels and induction of ET and JA biosynthesis marker genes expression for JA/ET (*LOX* and *ERF* genes) pathways in luffa plants pre-inoculated with ThM9 and TvA1 even in the absence of DM pathogen inoculation in comparison to control, further supported the previous evidence of shear indication of *Trichoderma*-induced systemic (TISR) response that is effective in resisting the *P. cubensis* attack in luffa. Besides ISR, fortunately, root-associated mutualistic microbes, namely *Trichoderma* sp., such as *T. asperellum, T. brevicompactum, T. hamatum, T. harzianum*, and *T. virens*, not only impact plant nutrition and growth but also further boost plant defenses through systemic acquired resistance (SAR) as well against pathogens [75].

SAR is known to depend on the SA signaling pathway along with *nonexpressor of pathogenesis-related genes 1* (*NPR1*)-mediated induction of pathogenesis-related protein (PR) genes, including *PR1, PR2*, and *PR5*, while ISR is triggered by the JA- and ET-signaling pathway with involvement of *NPR1* without *PR1, PR2*, and *PR5* gene induction [76,77]. However, this conventional understanding of ISR looks to be more complicated since the nature and composition of ISR are substantially influenced by the tripartite combination of plant BCA pathogen, and the overlap between SAR and ISR may be considerably more than the stated essential marker *NPR1* [78]. *T. asperellum* SKT-1-mediated SA biosynthesis and systemic resistance induction against cucumber mosaic virus in *A. thaliana* have been explored by [79].

Plant species are known to adopt either of the two pathways for SA biosynthesis, including isochorismate synthase (ICS) and phenylalanine ammonia-lyase (PAL) pathway [80], however, *Glycine max* has shown equal involvement of ICS and PAL pathway in its SA production. Shine [81] found a threefold increase in SA upon infection with *P. sojae* or *P. syringae* pv. *glycinea* (*Psg*), while silencing either the PAL or the ICS pathway resulted in significantly lower levels of SA accumulation during pathogen infection and increased susceptibility to infection by either of these pathogens, and silencing both pathways resulted in significantly increased susceptibility to infection by either of these pathogens. PAL enzyme is involved in the biosynthesis of SA as an essential signal involved in plant systemic resistance [82,83]. It is induced by wounding, pathogen infection, nutrient depletion, extreme temperatures, and ultraviolet (UV) irradiation [69,84]. The current study also showed a significantly increased level of SA accumulation and elevated PAL gene expression in luffa plants pre-inoculated with ThM9 and TvA1 with and without DM infection in comparison to control plants, which is a shear indication of *Trichoderma*-induced SAR’s ability to restrict the *P. cubensis* attack in luffa.

Furthermore, in pearl millet, the elicitation of systemic immunity against DM has been reported to be associated with *T. hamatum*-induced modulation of physicochemical and molecular properties [22], such as callose-deposition, cell-wall lignification, and SA-inducible genes induction for defense enzymes (peroxidase, hydroxyproline-rich glycoproteins, and polyphenol oxidase), which proves a connection between SA-accumulation and *Trichoderma*- elicited systemic resistance.

Chitin and β-1,3-glucan are major components of the fungal cell wall [85,86], while chitinases and β-1,3-glucanases lytic enzymes synthesized by *Trichoderma* promote their mycoparasitic activity for pathogen cell wall disintegration [87]. In addition, other cell-wall-degrading enzymes including those hydrolyzing minor polymers such as β-1,6-glucans further ensure the complete and effective disintegration of fungal mycelial or conidial walls by *Trichoderma*. A chitin-induced subtilisin-type serine proteinase was previously depicted in a *T. harzianum* mycoparasitic strain [88]. The β-1,6-glucanases have also been reported to degrade cell walls in yeast, filamentous fungi (Yamamoto, Kobayashi, and Nagasaki, 1974), and bacteria [89].

Proteome and secretome analysis of *T. harzianum* grown in the presence of *F. solani* or *B. cinerea* cell walls showed elevated proteins related to mycoparasitism (chitinases, 1,3-glucanases, glucoamylases, and proteases), suggesting that the pathogen cell wall is the main target of *T. harzianum* in the biocontrol mechanism [90]. Our study also revealed the higher lytic enzyme activities along with the higher gene expression of *Chitinase, β-13-Endoglucanase*, and *Subtilisin-like protease* of ThM9 and TvA1 endophytic fungi upon confrontation with *P. cubensis* pathogen. These findings are suggestive of the biocontrol mechanism governed by ThM9 and TvA1 endophytic fungi against DM of luffa. Upon pathogen attack, the plant initiates a passive defense response in the form of antimicrobial compounds production or active defense induced by the pathogen itself. In passive defense, the accumulation of defense-related enzymes in plants immunizes them before the attack of the pathogen. The antioxidant defense system comprises enzymes (such as SOD, POX, CAT, and APX), involved in the defense of plants in plants by protecting them against oxidative damage either by suppressing the ROS production or by scavenging the already-produced ROS [91,92,93]. POD participates in phenolic compounds biosynthesis and lignification, which are effective against pathogens. breakdown of hydrogen peroxide and ethylene production is also activated by PODs to induce a defensive response. Its role as oxidoreductase is well-known in defense mechanisms, lignin biosynthesis, hormone regulation, and indoleacetic degradation [94]. The current study also revealed an induction in gene expression of *Peroxidase* in ThM9- and TvA1-pre-inoculated luffa seedlings compared to control. Catalase enzyme decomposes the hydrogen peroxide into water and oxygen. H_2_O_2_ plays an important role in triggering hypersensitive cell death. It induces cell-protectant genes in surrounding cells of plants, thus limiting the spread of cell death. Increased level of H_2_O_2_ always correlates with the stimulation of disease resistance in plants. Consistently, our study also showed over-accumulation of H_2_O_2_ upon pathogen attack but the reduction in ThM9- and TvA1-pre-inoculated seedlings compared to control, indicating hypersensitive response (HR) or systemic acquired resistance in plant tissues in the form of localized H_2_O_2_ toxic for the pathogen. Moreover, SOD antioxidant enzyme is also involved in plant defense response by converting very reactive and toxin ROS (superoxide anion, hydroperoxyl radical, and singlet oxygen) to the less toxic molecule (hydrogen peroxide) [95]. Our study showed that the *catalase 2, superoxide dismutase 3*, and *L-ascorbate peroxidase* gene (*ROS scavenger enzyme*) showed an increase in ThM9 and TvA1 pre-inoculated seedlings compared to control. Phenolic compounds produced upon pathogen attack are toxic to the pathogen but beneficial for plants, as these are consumed for plant growth, development, and reproduction. Previously, it was known that total soluble phenols in cucumber leaves increased significantly with the applications of elicitors. Consistently, our study also showed that gene expression of *polyphenol oxidase* enzyme for *polyphenol biosynthesis enzyme* was induced in ThM9- and TvA1-pre-inoculated seedlings compared to control, indicating the active catabolism of phenolics. SA-induced elicitation mechanism for resistance against pathogens is vital not only for the expression of PR genes but also regulates the production of defense-related compounds such as callose, lignin, and defense enzymes linked with both local and systemic acquired resistance. Defense-related genes play a crucial role in DM resistance. The modulation in gene expression of PR proteins and other crucial defense enzymes participating in the *Trichoderma*-induced ISR and SAR in luffa were evaluated. The previous report also showed that endophytic *T. hamatum* elicited systemic immunity against DM disease of pearl millet through induction of defense proteins (PR5, PR1, hydroxyproline-rich glycoproteins; HRGPs) and defense enzymes (peroxidase, phenylalanine ammonia-lyase, β-1,3-glucanase, and polyphenol oxidase (PPO). Moreover, pathogenesis-related proteins, including *PR-5, PR-1*, and β-1,3-glucanase (*PR-2*), are often used as marker genes for the SA-dependent SAR, and basic chitinase (*PR-3*), plant-defense 1.2 gene (*PDF1.2*), and pathogenesis-related 4 (*PR-4*) are used as marker genes of the JA-dependent SAR [18]. Therefore, in-depth molecular mechanisms involving potential genes, proteins, and metabolites associated with DM resistance must be explored to enhance the disease resistance of luffa that recently has been sequenced at the whole-genome level [96]. *PR* gene expression regulation is a delicate mechanism for plant survival and growth upon pathogen attack that is intimately controlled by the involvement of signaling components such as hormones (SA, JA, and ET) [97,98,99,100]. The current study also showed the enhanced expression of antimicrobial enzymatic genes, phytohormonal biosynthesis, and signaling gene and pathogenesis-related (PR) proteins genes. For example, *lipoxygenase* (*LOX*) gene (*JA biosynthesis enzyme*) expression was increased in ThM9- and TvA1-pre-inoculated seedlings compared to control. *Ethylene-responsive transcription factor* gene expression has induced an increase in ThM9- and TvA1-pre-inoculated seedlings compared to control. Phytoene synthase (PSY) catalyzes the first rate-limiting step in carotenoid biosynthesis to control the overall carbon flow to carotenoid production. Carotenoids are precursors of ABA (hormonal growth regulator and signaling molecule) that trigger and amplify the abiotic stress resistance responses in plants by activating the synthesis of its precursors by inducing PSY gene expression. Current research showed that the gene for *9-cis-epoxycarotenoid dioxygenase* (*NCED2*) enzyme for ABA biosynthesis also showed increased expression in ThM9- and TvA1-pre-inoculated luffa seedlings compared to control with or without pathogen inoculation. The gene for *zeaxanthin epoxidase* (*ZEP*) enzyme that biosynthesizes the precursor (violaxanthin) for ABA production exhibited higher expression in ThM9- and TvA1-pre-inoculated seedlings compared to control with or without pathogen inoculation. Our study also showed induction in the expression of defense-related genes (*MLP3; major latex-like protein*) and *abscisic acid receptor* gene in luffa plants pre-inoculated with ThM9 and TvA1 with and without DM infection in comparison to control plants indicating a role of ABA-induced stress tolerance. Previously, it was known that major latex-like protein 43 (MLP43) acts as a positive regulator during ABA responses to initiate tolerance against drought in *A. thaliana* [101]. In plants, systemic response induction is connected with the effect of SMs acting as antimicrobial compounds secreted by *Trichoderma* [102]. Steroid derivatives are involved in the signaling mechanisms and maintenance of the ISR status in long-distance tissues. Previous reports have shown that terpenes act as signals related to pathogen attacks that control the steroidal metabolism mediated by *Trichoderma* in plants [103]. The transition of (S)-2,3-epoxysqualene into cycloartenol is mediated by cycloartenol synthase enzyme, which is a crucial biochemical reaction controlling the production of steroid-based SMs in the peak state of defense in plants. Cycloartenol is an important triterpenoid of the sterol class found in plants and is the starting point for the synthesis of most plant steroids [104]. Recently, it has been reported that the cycloartenol synthase (CAS1) proteins are involved in the biosynthesis of cycloartenol (an important plant steroid belonging to the triterpenoid of the sterol class), which is essential for plant cell viability [105]. *Cycloartenol synthases* have been cloned from angiosperm plants such as *A. thaliana, Pisum sativum, L. cylindrica*, *P, ginseng*, and *G. glabra* [106,107]; the slime mold *D. discoideum* [108], and the bacterium *S. aurantiaca* [109]. Our results showed induction in *cycloartenol synthase* (*CAS1*) gene expression in ThM9- and TvA1-pre-inoculated luffa seedlings compared to control, indicating the initiation of the steroid-based secondary metabolism for defense-related active signaling mechanism in luffa against DM pathogen. Moreover, the polyubiquitin genes also are known to be involved in different fungal virulence pathways in plants. In plants, polyubiquitins have a crucial role in controlling protein turnover in abiotic stress responses for stress-tolerance induction. Polyubiquitins also influence stress regulation through ROS homeostasis. These genes are also involved in the induction of innate immune responses in plants upon pathogen attack. Since plants depend on innate immune responses (SAR and hypersensitive responses), it is well-recognized that every step of the innate immune mechanism, starting from pathogen recognition to response, is under the ubiquitin–proteasome system (UPS)-mediated regulation. Plants have the adopted the UPS switches to activate PR proteins and SA-mediated SAR [97]. Consistently, our study also revealed the higher expression of the *polyubiquitin* gene in luffa plants pre-inoculated with ThM9 and TvA1 with and without DM infection in comparison to control plants, indicating the involvement of SA-induced stress tolerance governed through the effector-triggered immunity (ETI) signaling cascade. Moreover, *phenylalanine ammonia lyase* (phenol and SA biosynthesis key enzyme) gene expression was also induced in luffa plants pre-inoculated with ThM9 and TvA1 with and without DM infection in comparison to control plants, further confirming the biosynthesis boost of SA and its role in SAR. Furthermore, two separate routes for the detection of pathogens have been recognized so far: pathogen-associated molecular patterns (PAMP)-triggered immunity (PTI), which involves receptor proteins to detect pathogenic proteins such as flagellum, and effector-triggered immunity (ETI), which directly detects effector (avirulence) proteins released by a pathogen [110]. The plant immune system has developed a checkpoint regulatory route to maintain the homeostasis of these receptors to effectively respond to pathogens without causing an autoimmune response. PTI receptors, such as flagellin-sensitive 2 (FLS2) and lysm-containing receptor-like kinase 5 (LYK5), are constantly degraded by a UPS-mediated system involving PUB13 E3 ligase in normal growth conditions to prevent constitutive or autoimmune [111], while ETI receptors (resistance (R) proteins) also function as the second line of defense against pathogens. The largest group of plant R genes is *nucleotide-binding site–leucine-rich repeat* genes, including *suppressor of NPR1 constitutive 1* (*SNC1*). It is also known that E4 ubiquitin-conjugating factor (MUSE3) negatively regulates the NLR-receptors such as suppressor of NPR1 constitutive 1 (SNC1) for controlling constitutive immune response [112]. The current study also showed that the *NBS-LRR* gene was induced in ThM9- and TvA1-pre-inoculated seedlings compared to the control. Upon consortial pre-treatment with ThM9 and TvA1, a 2.5-fold increase was noticed for *NBS-LRR-resistance protein* gene expression compared to control with and without DM infection in comparison to control plants, indicating the trigging of ETI response against the pathogen in luffa for biotic stress tolerance.

## 5. Conclusions

The current research exposed the potential of endophytic ThM9 and TvA1 fungi isolated from *C. indica* host plants as effective biostimulators and biotic stress-tolerance ameliorators for luffa plants. The consortial inoculation of ThM9 and TvA1 was evidenced in promoting the growth rate and biomass by producing growth-promoting phytohormones and metabolites along with improved photosynthetic activity, optimized stomatal conductance, and stabilized cellular membrane integrity in luffa.

**Figure 10:**
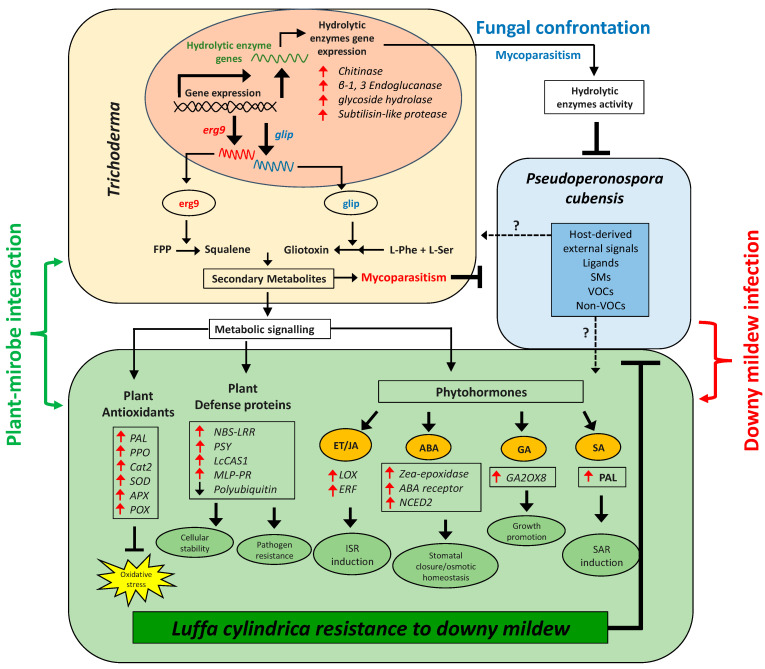
Model of ThM9 and TvA1 action upon *P. cubensis* infection in luffa.

The consortial inoculation of ThM9 and TvA1 fungi was also evinced to be an effective mycoparasitic fungal isolate and efficient biocontrol agent against DM disease of luffa. The growth-promoting ThM9 and TvA1 endophytic fungi elicited the systemic immunity against luffa DM disease. In summary, this study provides evidence that upon exposure to DM pathogen (*P. cubensis*), ThM9 and TvA1 pre-inoculation alleviated disease by reducing the pathogen proliferation through boosted production of anti-pathogenic metabolites, lytic and antioxidant enzymes, and defense-related proteins and inducing ABA, SA, JA, and ET biosynthesis and signaling gene expression for triggering SAR and ISR defense mechanisms in luffa (Figure 10). Altogether, our study discloses the complexity of the plant’s response towards beneficial fungal microbes, also demonstrating the positive role of ThM9 and TvA1 that ably activated the defense mechanisms in luffa against DM in a differential manner.

## Figures and Tables

**Table 1:** Primers used for PCR and RT-qPCR analysis.

Gene Accession	Gene Description	Primer Sequences
***P*. ** ***cubensis*** **Primers for PCR analysis**		
*JF414553.1*	*Cytochrome c oxidase subunit II* (*cox2*)	*F-TAATTGTAGTTACAGTATTC*
		*Clade 1 R-GTAATTAATACTCGAATATGG*
		*Clade 1/2 R GTAAAACATCAGAAGCTGTG*
***T. virens* Primers for PCR analysis**		
*JN039096.1*	*Translation elongation factor 1-like* (*tef1*)	*F-ACACAGCTAACCATTCGCCA*
		*R-TCCTTGGTTTAGCACTGGGC*
***T. harzianum* Primers for PCR analysis**		
*MW407164.1*	*RNA polymerase II* (*RPB2*)	*F-GCAGGAAGATGACCCGGAAA*
		*R-TGGAAGGGTGGACAACATGC*
***T. virens* Primers for RT-qPCR**		
*KP641615.1*	*Chitinase* (*ech42*)	*F-GATGACACTCAGGCCACCAA*
		*R-TGGCAAACAAGTTGGCATCG*
*MG702349.1*	*Endoglucanase* (*EG4*)	*F-GGTCTCATCAGTGGCGGAAA*
		*R-AATGAGTTCGTGGCGTAGCA*
*XM_014103795.1*	*Subtilisin-like protease*	*F-CATCCGATACCGAGCACGAA*
		*R-TGGCCTTGGTTCCAAAGGTT*
*NW_014013747.1*	*Cellulase/glycoside hydrolase*	*F-CCAGCGAGCATAGTTGTGGA*
		*R-ACTCTGGACGCCACCAATTT*
*XM_014100723.1*	*Non-ribosomal peptide synthetase GliP*(*NRPS dioxopiperazine synthetase*)	*F-CAAGGAAAACTTGTGGGCCG*
		*R-AGGGAGGAGAGCTGGTAGTG*
*FJ442590.1*	*Actin* (*ACT*)	*F-GTCCTTGGTCTTGAGAGCGG*
		*R-GAATGCAATTAGCGCCACTG*
***T. harzianum* Primers for RT-qPCR**		
*MG601052.1*	*Chitinase*	*F-TAACTACTCCAAGCTGCGCC*
		*R-GAATCGGTGTTGAAGGGGGT*
*MG702349.1*	*Endoglucanase* (*EG4*)	*F-GGTCTCATCAGTGGCGGAAA*
		*R-AATGAGTTCGTGGCGTAGCA*
*KC876057.1*	*Subtilisin-like serine protease*	*F-CATCAACGACGTCCAGACCA*
		*R-TCTTGGTTCTCGTTACCGGC*
*NW_020209251.1*	*Cellulase/glycoside hydrolase*	*F-TGTCATGGGGTAGCAAAACGA*
		*R-ACTCGATGTGGAAGACAGGC*
*XM_024916014.1*	*Squalene synthase* (*SQS1/ERG9*)	*F-TGGAATGGCAACAAAGTTGA*
		*R-AAGGAGATTTGGAGCAAGCA*
*FJ442452.1*	*Actin* (*ACT*)	*F-TGTCCTTGGTCTTGAGAGCG*
		*R-CGTAAAGCTGTGCGACTCAAT*
***Luffa*** ***aegyptiaca* Primers for RT-qPCR**		
*MN548044*	*Elongation factor 1α* (*EF-1α*)	*F-TCAAGAAGGTCGGATACA*
		*F-ACAGGGACAGTTCCAATAC*
*KP341758.1*	*Phenylalanine ammonia-lyase; PAL*	*F-CTGGGTGATGGAGAGCATGA*
		*R-CTCTTGTTGCTGAGTGAGGC*
*KM506755.1*	*Peroxidase; POD*	*F-CGCTCTATCTGGGGCACATA*
		*R-AATGTGTCCGGTGTTGTTGG*
*KR819890.1*	*Polyphenol oxidase* *; PPO2*	*F-AAGACGTCGCAGCTCAAATC*
		*R-CGTGCCATCATCAAGAACGT*
*KR184674.1*	*Catalase 2; CAT2*	*F-GCGATTTGTGGAAGCGTTAT*
		*R-ATCCAATCACGTTGGCTTTC*
*KX092448.1*	*Superoxide dismutase 3; SOD3*	*F-CCGAGCAGAGTGTTTTGTGA*
		*RACCCTACAGGGGGATTTGAC*
*KX092439.1*	*L-ascorbate peroxidase; APX*	*F-GGGGTTGTTGCTGTTGAAGT*
		*R-GATTGGTTGTCCAAGCACCT*
*KX092434.1*	*Peroxidase 5; POD5*	*F-CCCCTGGAGTTGTTGCTTTA*
		*R-GCAACCCCTTGTTGTCAAGT*
*MF678593.1*	*Gibberellin 2-beta-dioxygenase 8-like protein; GA2OX8* (*Gibberellin 2-beta-dioxygenase 8—Arabidopsis, involved in the pathway gibberellin biosynthesis*)	*F-GCGGATTCCAACAGAGAGAG*
		*R-CGGTAATCAGCCAATCTGGT*
*MF678591.1*	*Ethylene-responsive transcription factor; ERF*	*F-CCGGAGCTGGTAAAATCAAA*
		*R-CTCGTGGGGTTTGAAAAAGA*
*KX092444.1*	*Lipoxygenase; LOX* (*jasmonic acid biosynthesis*)	*F-ATTCGGGGTAATGGAAAAGG*
		*R-GGAACATAAACCGGATGTGG*
*MK649987.1*	*Zeaxanthin epoxidase; ZEP* (*ABA biosynthesis enzyme*)	*F-AACCGACCGAGTTTATCACG*
		*R-AATCCAGCGGAGGAAAGATT*
*KX092441.1*	*9-cis-epoxycarotenoid dioxygenase; NCED2* (*ABA biosynthesis*)	*F-AAGACGATTTGCCGTACCAC*
		*R-TTCGAGGTCTTTGGATTTGG*
*MF678592.1*	*Abscisic acid receptor*	*F-GTCGTTTGTTGTGGATGTGC*
		*R-TGGAAAAATCGATTGGGAAG*
*LC177373.1*	*Major latex-like protein 3; MLP3* (*positive regulator of ABA responses and induces drought tolerance and pathogen resistance*)	*F-TTTCGGGGATTTGGTCAATA*
		*R-GGCTTCACCAGATCTCCATC*
*JN230655.1*	*NBS-LRR resistance protein gene* (*pathogen resistant*)	*F-GGCAAGAAGTCGTGATTGGT*
		*R-CTAAGGGCAATCCTCCACAG*
*KX092450.1*	*Phytoene synthase; PSY* (*phytoene synthase is a transferase enzyme involved in the iosynthesis of carotenoids*)	*F-TTCAAATTCCCGATTCTTCG*
		*R-TTCACTAGAGCCGCCTGTTT*
*AB033334.1*	*LcCAS1 mRNA for cycloartenol synthase* (*biosynthesis of steroids*)	*F-AATCCACTTCCTCCCGAACT*
		*R-CACACTCATTGCGAGCCTTA*
*KR349345.1*	*Polyubiquitin* *; Ubi*	*F-GTCAACCCTCCACCTTGTGT*
		*R-TCCAGCGAAGATCAACCTCT*

**Table 2:** Effect of ThM9 and TvA1 endophytes on seed germination and seedling vigor index of luffa. Seedling vigor index was calculated on percentage germination and mean root and shoot lengths of the seedlings. The values are mean from three experiments and designated with the different letters show significant differences according to Tukey’s HSD test at *p* = 0.05.

Inoculations	Inoculum Concentration	Germination (%)	Seedling Vigor
**Control**	Non-inoculated	84 ± 2.013 ^a^	1865 ± 2.013 ^a^
**ThM9**	1 × 10^8^ CFU mL^−1^	88 ± 2.014 ^ab^	1895 ± 2.013 ^ab^
**TvA1**	1 × 10^8^ CFU mL^−1^	89 ± 2.014 ^ab^	1896 ± 2.013 ^ab^
**ThM9 + TvA1**	(1 × 10^8^ CFU mL^−1^) + (1 × 10^8^ CFU mL^−1^)	97 ± 2.014 ^b^	1983 ± 2.013 ^b^

**Table 3:** Endophytic efficacies against cucumber DM tested by sporangia-releasing inhibition assay. * Different letters represent significant differences.

Inoculations	Releasing Ratio (%)	Releasing Inhibition Ratio (%) *
**Control**	89.3	---
**ThM9**	22.6	79.1 ^a^
**TvA1**	24.3	81.4 ^b^
**ThM9 + TvA1**	11.1	95.1 ^c^

## Data Availability

Dataset for sequencing of the Internal Transcribed Spacer (ITS) of 18S rDNA for TvA1 and ThM9, performed in the present study, can be found in the online repository (https://www.ncbi.nlm.nih.gov/genbank/). Sequences were submitted to NCBI GenBank under accession no. ON315869 (accessed on 22 April 2022) for *T. harzianum* (ThM9), accession no. ON315868 (accessed on 22 April 2022) for *T. virens* (TvA1), and accession no. ON243884 (accessed on 15 April 2022) for *P. cubensis* (DM1).

## Supplementary Materials

The following supporting information can be downloaded at: https://www.mdpi.com/article/10.3390/jof8070689/s1, Figure S1: Molecular identification of fungal endophytes (A) M9; (B) A1 and fungal pathogen; (C) DM1; (D) specie identification for M9, A1, and DM1 using gene-specific primers; and (E) in vitro compatibility test for M9 and A1 endophytic fungi.
