# Supplementary material for: Immunomodulatory Molecular Mechanisms of Luffa cylindrica for Downy Mildews Resistance Induced by Growth-Promoting Endophytic Fungi
Source: J Fungi (Basel). 2022 Jun 29;8(7):689. doi: 10.3390/jof8070689 (PMC9324744; doi:10.3390/jof8070689)
Supplement: Supplementary file 1 [file jof-08-00689-s001.zip › jof-1798080-supplementary.pdf]

Journal of Fungi

MDPI

Supplementary Figure 1

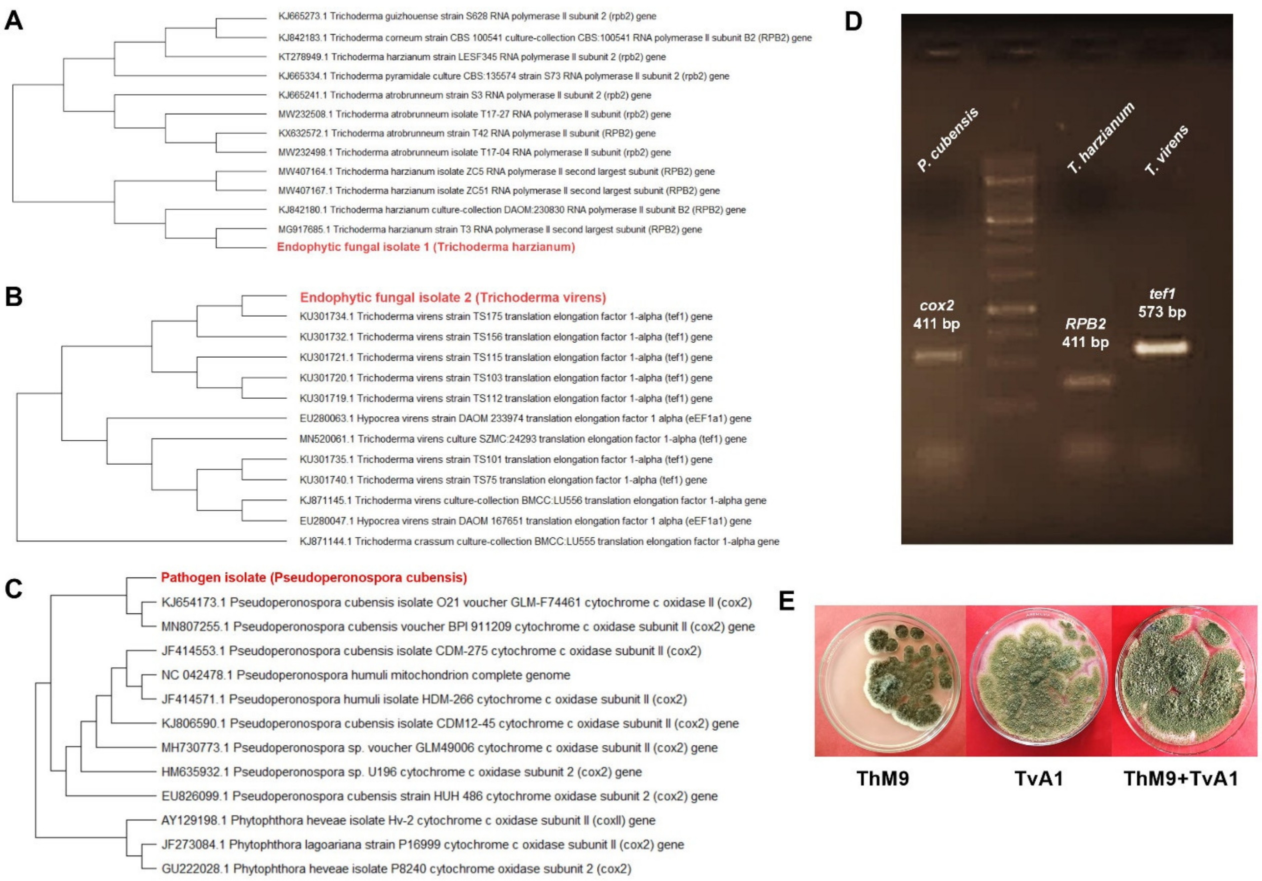

Supplemental Figure S1. Molecular identification of fungal endophytes (A) M9 (B) A1 and fungal pathogen (C) DM1 (D) specie identification for M9, A1, and DM1 using gene-specific primers (E) *In vitro* compatibility test for M9 and A1 endophytic fungi.
